# Bone Morphogenetic Protein 15 Knockdown Inhibits Porcine Ovarian Follicular Development and Ovulation

**DOI:** 10.3389/fcell.2019.00286

**Published:** 2019-11-19

**Authors:** Yufeng Qin, Tao Tang, Wei Li, Zhiguo Liu, Xiaoliang Yang, Xuan Shi, Guanjie Sun, Xiaofeng Liu, Min Wang, Xinyu Liang, Peiqing Cong, Delin Mo, Xiaohong Liu, Yaosheng Chen, Zuyong He

**Affiliations:** State Key Laboratory of Biocontrol, School of Life Sciences, Sun Yat-sen University, Guangzhou, China

**Keywords:** BMP15, transgenic pig, shRNA, follicular development, ovarian development

## Abstract

Bone morphogenetic protein 15 (*BMP15*) is strongly associated with animal reproduction and woman reproductive disease. As a multifunctional oocyte-specific secret factor, BMP15 controls female fertility and follicular development in both species-specific and dosage-sensitive manners. Previous studies found that BMP15 played a critical role in follicular development and ovulation rate in mono-ovulatory mammalian species, especially in sheep and human, but study on knockout mouse model implied that BMP15 possibly has minimal impact on female fertility of poly-ovulatory species. However, this needs to be validated in other poly-ovulatory species. To investigate the regulatory role of BMP15 on porcine female fertility, we generated a BMP15-knockdown pig model through somatic nuclear transfer technology. The BMP15-knockdown gilts showed markedly reduced fertility accompanied by phenotype of dysplastic ovaries containing significantly declined number of follicles, increased number of abnormal follicles, and abnormally enlarged antral follicles resulting in disordered ovulation, which is remarkably different from the unchanged fertility observed in BMP15 knockout mice. Molecular and transcriptome analysis revealed that the knockdown of *BMP15* significantly affected both granulosa cells (GCs) and oocytes development, including suppression of cell proliferation, differentiation, and follicle stimulating hormone receptor (*Fshr*) expression, leading to premature luteinization and reduced estradiol (E2) production in GCs, and simultaneously decreased quality and meiotic maturation of oocyte. Our results provide *in vivo* evidence of the essential role of BMP15 in porcine ovarian and follicular development, and new insight into the complicated regulatory function of BMP15 in female fertility of poly-ovulatory species.

## Introduction

In the past three decades, increasing studies have revealed the important role of the oocyte-specific secreted factor, bone morphogenetic protein 15 (BMP15), in mammalian ovarian and follicular development through its multiple functions including promoting granulosa cells (GCs) proliferation and steroidogenesis (Otsuka et al., 2000, 2001; Moore et al., 2003; Moore and Shimasaki, 2005), preventing cell apoptosis and premature luteinization (Hussein et al., 2005; McNatty et al., 2005; Juengel et al., 2011; Chang et al., 2013; Zhai et al., 2013), regulating glycometabolism and lipid metabolism (Sugiura et al., 2007; Su et al., 2008), and controlling oocyte competence and ovulation (Fabre et al., 2006; Hussein et al., 2006). As a key signaling molecule mediating the dialogue between oocyte and its surrounding somatic cells (Gilchrist et al., 2008), BMP15 is expressed initially in the early follicle stage, and the expression gradually increases in subsequent follicle stages till the period of ovulation and/or luteinization (Paradis et al., 2009; Sun et al., 2010). This expression pattern differs with species, for example, the initial expression of BMP15 protein can be found in primary follicle (PF) stage of sheep, human, and pig, but not until the pre-ovulatory stage in mice (Paulini and Melo, 2011). BMP15 protein is secreted as BMP15/BMP15 homodimers and BMP15/growth differentiation factor 9 (GDF9) heterodimers, and it binds to the membrane bound type II serine/threonine kinase BMP receptor (BMPR2) and type I activin receptor-like kinase ALK6, resulting in the phosphorylation and activation of the SMAD pathways (Liao et al., 2003; Pulkki et al., 2012; Mottershead et al., 2013). In particular, BMP15 homodimers bind to ALK6 receptor to activate the Smad1/5/8 signaling pathway in some species, for example, in humans and sheep but not in rodents. However, BMP15/GDF9 heterodimers bind to BMPR2 receptor to activate Smad2/3 signaling pathway in all reported species (sheep, human, mouse, pig, etc.) (Reader et al., 2011, 2016; Peng et al., 2013; Lin et al., 2014). In most cases, BMP15/GDF9 heterodimers are more potent than BMP15 homodimers in the regulation of GCs, oocytes, and zygote development (Peng et al., 2013).

Bone morphogenetic protein 15 mutation or deficiency has been associated with altered female fertility in different species. As previously reported, natural mutations in BMP15 of sheep can lead to increased ovulation rate and litter size in heterozygotes but infertility in homozygotes due to bilateral ovarian hypoplasia (Braw-Tal et al., 1993; Smith et al., 1997; Galloway et al., 2000; Fabre et al., 2006). Altered fertility has also been reported in sheep immunized with BMP15 mature protein or different region of the peptides (Juengel et al., 2002, 2004, 2013; McNatty et al., 2007). In humans, BMP15 mutations have been associated with primary ovarian insufficiency (POI) and infertility phenotype in women (Chand et al., 2006; Abir et al., 2014; Al-ajoury et al., 2015). However, in the poly-ovulatory mice, there was no significant difference between *BMP15*^±^ females and wild-type in terms of the ovulation rate, and only a mild reduction of fertility was observed in *BMP15* null female mice (Yan et al., 2001). Several studies have attempted to determine if there are species-specific differences in the BMP15 system that may play causal roles in the differences in fertility observed in poly-ovulatory mice and mono-ovulatory sheep and humans. One study has attributed the species-specific differences to the temporal variations in the production of the mature form of BMP15. The study found that mouse BMP15 mature protein was barely detectable until the pre-ovulatory stage, when it was markedly increased (Yoshino et al., 2006). Another study reported that defects in the production of mouse BMP15 mature protein correlate with species-specific differences (Hashimoto et al., 2005). Moreover, a phylogenetic analysis found a better conservation in areas involved in dimer formation and stability of BMP15 within mono-ovulatory species, but high variations in these areas within poly-ovulatory species, implying a correlation with altered equilibrium between homodimers and heterodimers, and modified biological activity that allows polyovulation to occur (Monestier et al., 2014). Hence, it seems that the role of BMP15 in the regulation of follicular development and ovulation rate was more critical in mono-ovulatory mammalian species than in poly-ovulatory animals. However, the role of BMP15 in ovarian and follicular development in poly-ovulatory mammalian species has remained unclear, as this has not yet been investigated in *in vivo* studies of non-rodent poly-ovulatory mammals.

In this study, we aim to investigate the function of BMP15 in female fertility and follicular development of non-rodent poly-ovulatory mammal by using a *BMP15* knockdown transgenic (TG) pig model. The TG gilts had decreased female fertility with disordered estrous cycle, significant reduced ovarian size and follicle number, higher ratio of abnormal follicles, and none corpus lutein formed before 365 days old. We found that knocking down *BMP15* can impair porcine follicle growth and cause dysovulation mainly by influencing oocyte quality and oocyte meiotic maturation, suppressing GCs proliferation and GCs functions, including inhibiting the expression of *Fshr* and E2 production, resulting in premature luteinization. These effects on follicular cell functions could finally lead to the absence of dominant follicle selection but appearance of abnormally enlarged antral follicles (AFs) with ovulation dysfunction in TG gilts. Our findings were evidently different from the unchanged fertility observed with *BMP15*^±^ mice, strongly suggesting the important role of BMP15 in non-rodent poly-ovulatory mammals, thus providing the basis for further investigation on the different regulatory role of BMP15 in mono-and poly-ovulatory mammals.

## Materials and Methods

### Construction of shRNA Expression Vectors and Evaluation of shRNA Interference Efficiency

**FIGURE 1:**
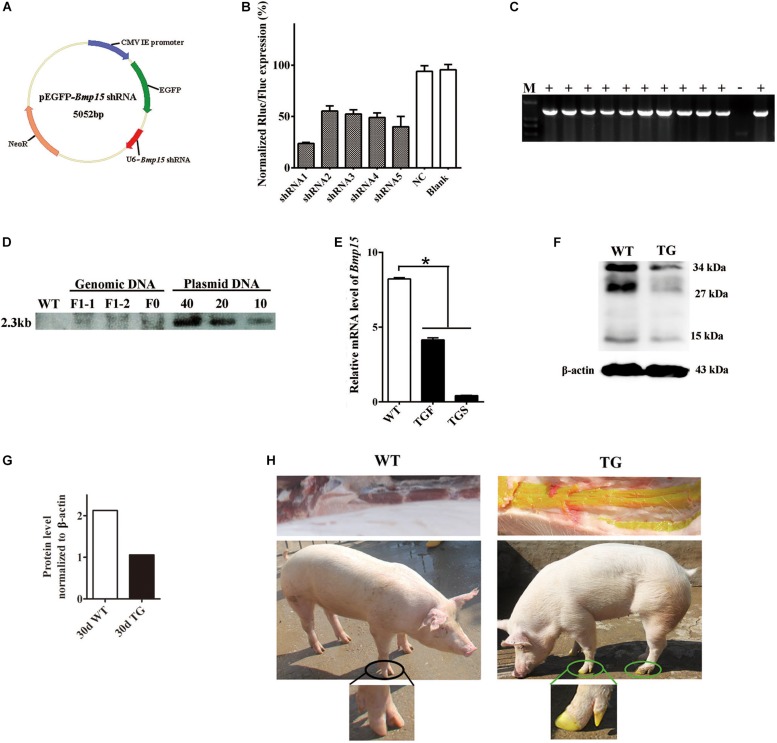
Generation of the *BMP15* knockdown pig model. **(A)** Diagram of shRNA expression vector. Synthesized *hU6-BMP15* shRNA fragment was inserted downstream of *EGFP* expression cassette on pEGFP-N1 vector. **(B)** RNA interference efficiency of five *BMP15* shRNAs was examined by a dual-luciferase reporter system after 48 h transfection of h293T cells. NC, random shRNA plasmid. **(C)** PCR analysis of the muscle tissue proved that the integrated *Bmp15* shRNA fragment had been transmitted to F1 gilts. +, TG gilt; -, sibling WT gilt; M, DNA Maker. **(D)** Southern blot analysis showed slightly less than 10 copies of constructed plasmids integrated in both F0 and F1 TG pigs, which was consistent with the result of about seven copies of qPCR analysis (data not shown). DNA with pEGFP-*Bmp15* shRNA plasmid copies of 10, 20, and 40 were used as the positive control. **(E)** qPCR analysis of *BMP15* mRNA level in 365 days old transgenic ovaries with two different phenotypes (TGF and TGS). TGF, transgenic ovary with many visible antral follicles on ovarian surface. TGS, transgenic ovary with streak phenotype. ^∗^*p* < 0.05. **(F)** Western blot analysis of BMP15 protein level in postnatal 30-day old TG ovaries. Three prominent, distinct bands were observed corresponding to apparent molecular weights of 34 kDa, 27 kDa, and 15 kDa. **(G)** Quantitative analysis of BMP15 protein levels based on the band intensity using Image J software. **(H)** F1 TG gilt showed a visible intense GFP fluorescence on the toes and muscle while subjected to sunlight.

Five shRNAs (Supplementary Table S1) targeting porcine *Bmp15* mRNA was designed and selected by Invitrogen’s web-based siRNA design software^1^. Human U6 promoter followed by each shRNA sequence was individually synthesized (Sangon Biotech, China) and cloned downstream of the EGFP expression cassette on pEGFP-N1 vector (Takara Bio, United States) to generate each pEGFP-*Bmp15-*shRNA expression vector (Figure 1A). Meanwhile, a scramble shRNA expression vector was generated as negative control. To evaluate the RNA interference efficiency of shRNA, porcine *Bmp15* CDS was synthesized (Sangon Biotech, China), and cloned into psiCheck II vector (Promega, United States) to generate psiCheckII-*Bmp15* plasmid. Each pEGFP-*Bmp15-*shRNA plasmid was respectively co-transfected with psiCheckII-*Bmp15* plasmid into HEK293 cells. After 48 h of culture, transfected cells were collected and subjected to RNA interference efficiency detection by using a dual-luciferase reporter system (Promega, United States). The shRNA with the most efficient RNA interference was selected for the generation of BMP15 knockdown pig model.

### Generation of *Bmp15* Knockdown Pig Model

Procedures for the generation of the *Bmp15* knockdown gilts are illustrated in Supplementary Figure S1. Briefly, the selected pEGFP-*Bmp15* shRNA plasmid was transfected into PEFs derived from a male Yorkshire pig. After G418 selection and fluorescence examination, EGFP-positive PEFs were used as donor cells for somatic cell nuclear transfer (SCNT). For SCNT, oocytes were collected from abattoir ovaries with a 20G needle connected to a syringe, and then cultured in HEPES-buffered tissue culture medium 199 and later maturation medium, until *in vitro* maturation. SCNT by handmade cloning and embryo transplantation were carried out by BGI Ark Biotechnology Company, China. After 114 days of pregnancy, we obtained two surviving F0 generation *Bmp15-*knockdown TG males. Next, we mated one TG boar with wild-type sows through artificial insemination (AI), and obtained F1 generation TG gilt for this study. Sibling gilts without pEGFP-*Bmp15*-shRNA integration were used as controls (WT) in this study. The protocol for the animal study was approved by the Institutional Animal Care and Use Committee (IACUC), Sun Yat-sen University (Approval Number: IACUC-DD-16-0901).

### Tissue Collection

A total of 54 animals including 25 WT and 29 TG gilts were sacrificed at ages of 30–500 days. Among these TG gilts, six gilts contained eight TGS ovaries at age of 110, 160, 200, and 365 days. Tissues of ovary, pituitary, muscle, liver, kidney, heart, and uterus were collected. Tissues used for RNA extraction were directly soaked in TRIzol reagent (Promega) and frozen rapidly in liquid nitrogen. Muscle tissues for DNA extraction together with the 30-day ovarian tissues for protein detection were directly frozen in liquid nitrogen before being transported to the laboratory. All the other ovaries were washed in sterilized saline water and photographed. Ovaries at the ages of 60 and 90 days (*n* = 6) were used for mRNA detection, and 365-day old ovaries (*n* = 6) of WT and TGF gilts were used for mRNA detection in ovarian tissues and isolated follicle, respectively. Primers for qPCR analysis are shown in Supplementary Table S3. Ten ovaries (5 WT and 5 TGF) at the age of 60–170 days were frozen in OCT and stored at −80°C before laser capture microdissection (LCM). Six ovaries (3 WT and 3 TGF) from different 365-day old gilts were used for dissection and follicular fluid collection. Furthermore, they were used for COCs collection and subsequent single-cell sequencing. The other ovaries (24 WT, 24 TGF, and 8 TGS) at age of 30–400 days were used for hematoxylin and eosin (HE) observation and immunohistochemistry (IHC) analysis; they were fixed in 10% (w/v) paraformaldehyde/0.02 M PBS (pH 7.2) on ice before transportation to the laboratory.

### Identification and Characterization of Transgenic Gilts

Transgenic pigs were first screened by GFP fluorescence on the toes under sunlight, and the integration of pEGFP-*Bmp15* shRNA plasmid in genome using genomic DNA extracted from muscle tissues was confirmed by PCR analysis (Supplementary Table S2). The copy number of integrated plasmid was determined by qPCR and Southern blot analysis. *Bmp15* mRNA expression level in TG pigs was detected by qPCR in 365-day old ovaries, and BMP15 protein level was detected by western blot analysis in 30-day old ovarian tissues.

### F1 Gilts Estrous Checking and Hormone Assays

About 50 F1 TG gilts at age of 170–400 days were checked daily for signs of estrous in the presence of an intact mature boar. TG and WT gilts (*n* = 2, each) at about 365 days old were chosen for daily vaginal smears analysis, and daily jugular venous blood collection at 9:00–11:00 AM for 24 days continuously. Vaginal cell smears analysis and estrous identification were performed as described in a previous report (Mayor et al., 2007). Daily blood samples were centrifuged at 1500 *g* for 15 min, and the serum samples were collected and stored at −80°C. These serum samples were thawed on ice prior to use in the quantification of the concentration of estradiol (E2) and progesterone (P4) by chemiluminescence immunoassay (CLIA) (Siemens, Germany).

### Histological Examination

Ovaries derived from gilts at age of 30–400 days were fixed in 10% (w/v) paraformaldehyde with 0.02 MPBS (pH 7.2) at 4°C for about 2 h. Next, they were cut into vertical slices of about 0.5 cm thickness and fixed in fresh 10% (w/v) paraformaldehyde for a cumulative period of 24 h. These slices were mounted in paraffin, and serially cut into 5 μm-thick sections by Rotary Microtome (MICROM, Germany), and stained with HE. Ovarian HE sections were observed and photographed under a fluorescent microscope (Zeiss, Germany).

Immunohistochemistry detection was performed by using the anti-Rabbit HRP-DAB Cell and tissue staining kit (R&D, CTS005) and anti-Goat HRP-DAB Cell and tissue staining kit (R&D, CTS008). Immunohistofluorescence examination was performed by using TSA plus Fluorescein (PerkinElmer, NEL741001KT) and Cyanine3.5 (PerkinElmer, NEL763001KT) kit. Antibodies of BMP15 (Eterlife, EL806306-100), GDF9 (Eterlife, EL901942-100), FSHR (Eterlife, EL912710), luteinizing hormone receptor (LHR) (Eterlife, EL904141), Caspase3 (Abcam, ab13847), 3βHSD (Abcam, ab154385), p-Smad1/5 (CST, 9516), Ki67 (Abcam, ab15580) and LC3B (Arigo, ARG55799) were diluted 1:100 with PBS. Other antibodies including ALK6 (Santa Cruz, sc5679), BMPR2 (Santa Cruz, sc5683), Smad2/3 (Santa Cruz, sc8332), Smad1/5/8 (Santa Cruz, sc6031R), and p-Smad2/3 (Santa Cruz, sc11769) were diluted 1:50 in PBS.

### Ovary Dissection and Follicular Fluid Collection

Each of the three 365-day TGF and WT ovaries derived from different individuals were flushed with sterilized saline water and placed in the incubator at 38°C during transportation to the laboratory. Later, visible AFs in these ovaries were dissected by scalpel blade and tweezers, and classified into three groups (1–3 mm, 3–5 mm, and >5 mm) according to their diameter, which was measured by a Vernier caliper. Total follicle number of each group was counted. Next, follicular fluid from AFs with diameter of 3–5 mm and diameter >5 mm were collected by a disposable 10 mL syringe. The concentrations of follicle stimulating hormone (FSH), LH, E2, and P4 in follicular fluid were quantified by the CLIA method (Siemens, Germany).

### Laser Capture Microdissection (LCM)

A total of 10 ovaries from WT and TGF gilts of age 60–170 days were embedded in OCT and placed on a cryostat (MICROM, HM560, Germany). All ovaries were cut into 7 μm-thick sections and mounted on RNAse free membrane slides (MMI, 50102). These membrane slides were then fixed in ice-cold 95% ethanol for 1 min, and later washed in 75% ethanol for 30 s. Subsequently, the sections were stained following a previously reported method (Golubeva et al., 2013). Briefly, the staining mixture was prepared with 1% cresyl violet in absolute ethyl alcohol, Eosin Y, RNAse free water, and 100% ethanol at the ratio of 3:1:4:4. Membrane slides were stained in this fresh staining mixture for 30 s and dehydrated thrice in 100% ethanol for 1 min, followed by 30 s incubation in xylene. Slides were finally dried for 5 min by a hair dryer blowing cold wind, and stored at −80°C until use.

The follicles were distinguished from each other as follows: PF was defined by a clear monolayer of cuboidal GCs; secondary follicle (SF) was defined by more than two layers of GCs but without any antrum; small antrum follicle (SAF) was defined by obvious small antrum but not completely separated granulose and cumulus cells; AF was characterized by a big single central antrum with completely separated granulosa and cumulus cell layers. The entire PF, SF, and SAF, but only parietal granulosa and theca cells of AF (APC) were isolated by LCM. Each type of the follicles of each ovary was dissected under 20 × magnification microscopic visualization using MMI Cell Cut Plus system (MMI, Swiss). Later, the dissections were treated with 100 μL of TRK Lysis buffer of the MicroElute total RNA kit (Omega) and 2 μL 2-mercaptoethanol. Both TGF and WT lysates were, respectively mixed according to their follicle stages after lysing for 10 min at 16°C, followed by storage on dry ice until RNA extraction. A total of eight LCM-derived RNA samples, PF^*WT*^, SF^*WT*^, SAF^*WT*^, APC^*WT*^, PF^*TGF*^, SF^*TGF*^, SAF^*TGF*^, and APC^*TGF*^, were used for transcriptomic analysis. RNA-seq was performed on an Illumina HiSeq 2000 using Illumina TruSeq SBS kit v2 (209 cycles including index) to obtain paired-end reads (2 × 100 bp).

### Single-Cell RNA Sequencing on Cumulus-Oocyte Complex (COCs)

Cumulus-Oocyte Complex were collected from large AFs (diameter, 5–7 mm) of 365-day old TGF and WT ovaries (*n* = 3) derived from different gilts by using a 20-gauge needle fixed to a 10 mL disposable syringe. They were pooled and placed on a stereomicroscope (Nikon). COCs with several layers of cumulus cells and uniform cytoplasm were selected. The selected COCs from both TGF and WT ovaries (*n* = 10 from each) were used for RNA micro-extraction by MicroElute total RNA kit (Omega). Total RNA was pre-amplified by SMARTer^®^ Ultra^TM^ Low RNA Kit (Clontech), and sequenced on Illumina HiSeq 2000 sequencing system.

### Data Analysis

All the mRNA detections were performed three times, and quantitative data are represented as the mean ± SEM. Measurements of hormones concentration were not repeated but were conducted on at least three individuals. Statistically significant differences were analyzed by *t*-test or one way analysis of variance, followed by Duncan’s *post hoc* test. A *p*-value < 0.05 indicated statistical significance.

For RNA-seq data analysis, raw RNA-Seq clean reads were obtained by removing reads containing low quality reads and/or adaptor sequences from raw reads and mapped to the pig genome (*Sus scrofa* 10.2), allowing up to two base mismatches. Differential expression analysis was performed using the Benjamini approach; genes with an adjusted *p*-value < 0.05 and log2 fold change >1 were designated as differentially expressed genes (DEGs). DEGs lists were submitted to the databases of Novogene Company (China) for further enrichment analysis. GO analysis was performed with Webgestalt software. In all the tests, *p* values were calculated using the Benjamini-corrected modified Fisher’s exact test, and *p* < 0.05 was taken as a threshold of significance. Venn diagrams were drawn using the web tool^2^. As determined by gene co-expression analysis, a correlation coefficient of 0.98 was set as the threshold value. Closely correlated genes were imported in cytoscape software to generate the co-expression network.

## Results

### Generation and Identification of *BMP15* Knockdown Pig Model

In our study, we generated a *BMP15* knockdown model through the shRNA technique. We designed and constructed five pEGFP-*BMP15-*shRNA plasmids, in which *BMP15* shRNA sequence was under the control of human U6 promoter (Czauderna, 2003) and inserted downstream of the *EGFP* expression cassette (Figure 1A). Each shRNA expression plasmid was co-transfected into HEK293T cell with a psiCheckII-*BMP15* plasmid to examine their RNA interference efficiency *in vitro* using a dual-luciferase reporter system (Boudreau et al., 2009). We found that shRNA1 was the most effective with a RNA inference efficiency reaching 76% (Figure 1B), and thus, this shRNA was selected for transfection into embryonic fibroblast cells (PEFs) derived from a male Yorkshire pig. Transfected PEFs were then subjected to G418 selection to select the cells with stable expression of EGFP as donor cells for SCNT (Baguisi et al., 1999; Polejaeva et al., 2000; Liu et al., 2019; Supplementary Figure S1A).

Both F0 and F1 TG pigs showed visible intense GFP fluorescence on the toes and muscle when subjected to sunlight (Figure 1H), directly suggesting that the pEGFP-*BMP15* shRNA plasmid was successfully integrated into the genome of F0 TG boar, and can be transmitted to the next generation through the germline. This was confirmed by PCR analysis of *Bmp15* shRNA fragment in the muscle tissue of F1 TG gilts (Figure 1C). The copy number of integrated plasmid was estimated to be approximately seven in F1 TG pigs as analyzed by the combination of qPCR using a *transferrin receptor* gene to normalize the genomic DNA (data not shown), and Southern blot analysis (Figure 1D). More importantly, evident decreased levels of *BMP15* mRNA (Figure 1E) in 365-day old TG ovaries and BMP15 protein in 30-day old TG ovaries (Figures 1F,G) strongly demonstrated the successful generation of a *BMP15* knockdown model, and implied an *in vivo* BMP15 knockdown efficiency of about 50% in TGF ovaries. Our qPCR data also revealed that *BMP15* mRNA was highly expressed in the ovary tissues, lowly expressed in the pituitary (Supplementary Figure S1B), but was undetectable in other porcine tissues (e.g., liver, muscle, and kidney).

### Knockdown of *BMP15* Was Associated With Disordered Reproductive Cycle of TG Gilts

**FIGURE 2:**
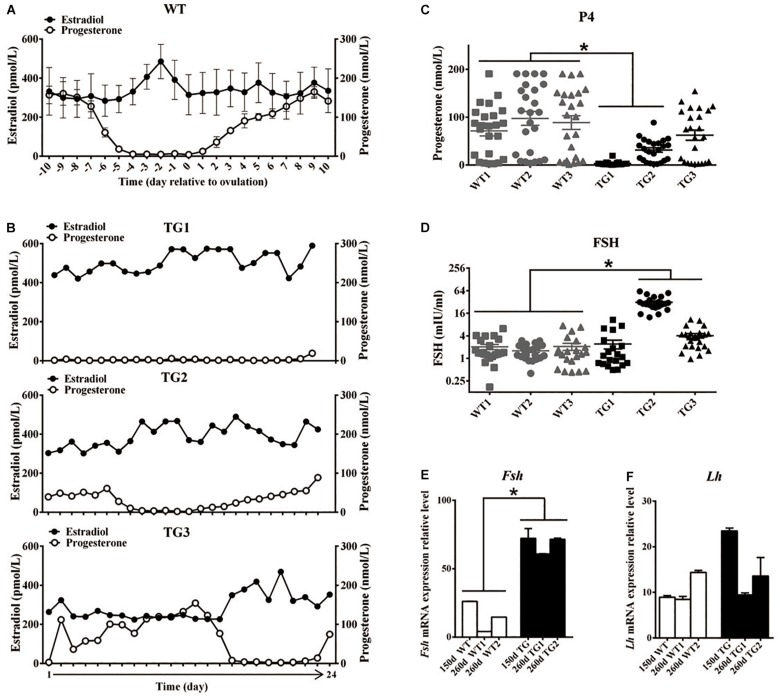
Transgenic gilts presented disordered estrous cycle and reproductive hormones. Plasma E2 and P4 concentration of 365-day old WT and TG gilts were measured at a 24-h interval for 24 days. **(A)** During the estrous cycle, representative WT gilts showed typical serum E2 concentration peak before ovulation, accompanied with marked decreased P4 concentration (*n* = 3), data are presented as mean ± standard deviation. **(B)** Irregular plasma E2 concentration peaks was observed in three representative TG gilts in a continuous 24-day measurement. **(C)** The average P4 concentration of two of the three TG gilts in a continuous 24-day measurement was significantly lower than that of WT gilts (*p* < 0.05). Each point stands for a P4 concentration value. **(D)** Two of the three 365-day TG gilts showed higher serum FSH concentration. Serum FSH concentration was measured at a 24-h interval for 24 days. **(E)** Expression level of *Fsh* mRNA in the pituitary of both 150- and 260-day old TG gilts were two-fold higher than that in WT gilts (*p* < 0.05). **(F)** The average level of *Lh* mRNA level in pituitary was not significantly different in TG and WT gilts. ^∗^Stands for statistical significance (*P* < 0.05).

All F1 TG gilts presented normal appearance and growth condition, and 50 of them at ages of 170–400 days were checked daily for signs of estrous in the presence of an intact mature boar. Surprisingly, we did not observe any obvious estrous behavior or vulvar appearance changes (e.g., increased redness, swelling, or mucus production) (Soede et al., 1994) in sexually mature TG gilts (Supplementary Figure S2A). About twenty gilts at ages of 240–400 days were bred by AI after treatment with PG600, but all failed to become pregnant. To determine if the disordered estrous cycle in TG gilts was related to reproductive hormonal changes, we measured the concentration of plasma estrogen (E2), progesterone (P4), and FSH in 365-day old gilts throughout the estrous cycle. The results showed that a typical peripheral E2 concentration peak before the onset of estrous can be observed in WT gilts, which is consistent with previous studies (Noguchi et al., 2010; Soede et al., 2011; Figure 2A). In contrast, irregular E2 concentration peaks were observed in TG gilts during a 24-day continuous measurement (Figure 2B). Cytological analysis of vaginal smears (Mayor et al., 2007) further proved that disordered estrous cycle occurred in TG gilts, as irregular cytologic changes was observed throughout a 16-day continuous examination (Supplementary Figure S2B). Furthermore, except for TG3 gilt, the average P4 level of the other two gilts was significantly lower than that of the WT gilts, with TG1 gilt containing bilateral asymmetric ovaries [one streak ovary (TGS ovary), and the other one TGF ovary] presenting the lowest serum P4 level. The other two TG gilts contain bilateral TGF ovaries (Figure 2C), and higher serum FSH concentration was found in TG2 and TG3 gilts (Figure 2D). In addition, we found over 2-fold up-regulated expression of *Fsh* mRNA level in the pituitary of both 150-day and 260-day old TG gilts (Figure 2E), but no significant difference in the expression level of *luteinizing hormone* (*Lh*) (Figure 2F). These results indicate a disordered reproductive cycle and potential ovarian dysfunction in TG gilts.

### Knockdown of *BMP15* Led to Inhibition of Follicular Development and Ovulation in TG Ovaries

**FIGURE 3:**
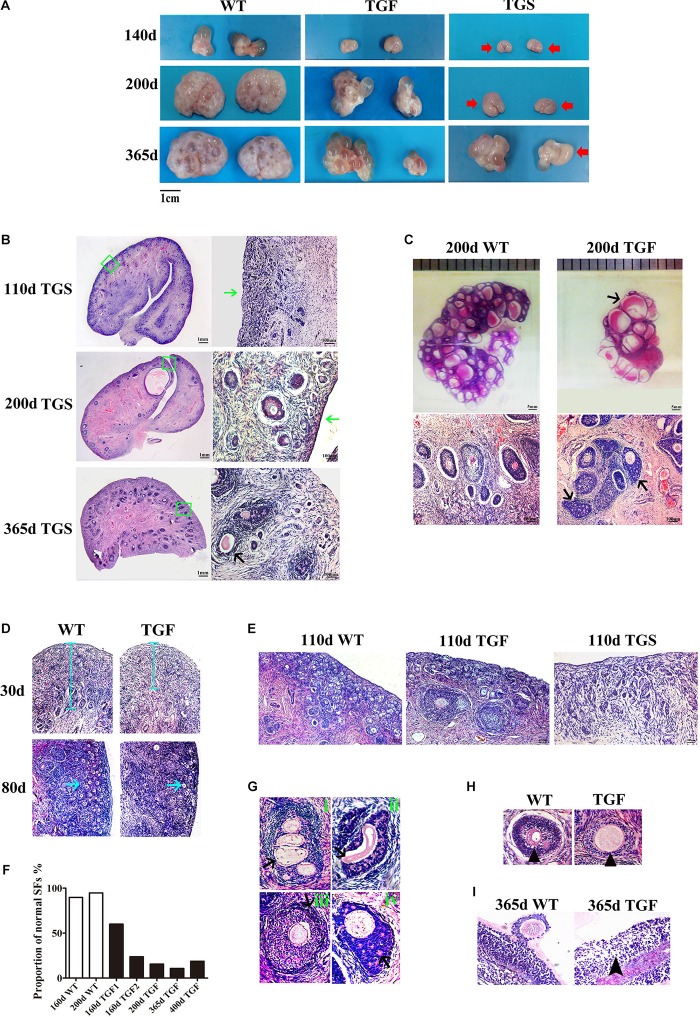
BMP15 knockdown-induced changes in ovarian and follicular development. **(A)** Representative photograms of ovaries collected from gilts of different ages showing reduced ovarian size and less visible follicles on the surface of TG ovaries as compared to ovaries from WT sibling. Bilateral TG ovaries were significantly different in size at ages of 200 and 365 days. Two ovarian phenotypes were found in TG ovaries, TGF ovaries had many visible large antral follicles on the ovarian surface. TGS ovaries contained none or less than three visible antral follicles (red arrows). **(B)** Histological observation of TGS ovaries showed that 110- and 200-day old TGS ovary presented major primary-like follicles sparsely scattered on the cortex (green arrows), while 365-day old TGS ovarian section was predominantly occupied by degraded secondary follicles (black arrow). **(C)** On the 200-day TGF ovarian section, decreased number of follicles and enlarged antral follicles (black arrow) was observed. In addition, the degradation of GCs in abnormally organized GC layer structure of secondary follicles was observed (black arrows). **(D)** In 30- and 80-day old TGF ovaries, drastically decreased number of early stage follicles led to thinner ovarian cortex (blue line and blue arrows). **(E)** Comparison of three ovarian phenotypes at the age of 110 days showed less number of early stage follicles in TGF ovarian cortex, and the minimum number of follicles in TGS ovaries. **(F)** Markedly reduced proportion of normal secondary follicles in the TGF ovaries. Secondary follicles in three sections of each ovary were counted. **(G)** Representative images of abnormal TGF secondary follicles (black arrows), including multiovular follicle with highly irregularly organized theca cell layers (i); follicle with oocyte-free structure, and abnormally thickened zona pellucida surrounded by highly degraded GCs (ii); follicle with abnormally thickened theca layers (iii); follicle with enlarged oocyte surrounded by highly irregularly organized GC layers with holes formed by degradation of GCs (iv). **(H)** TGF follicle showed larger oocyte in the early secondary follicle stage (black arrow head). **(I)** Smaller GCs were loosely organized in TGF antral follicles (black arrow head).

**FIGURE 4:**
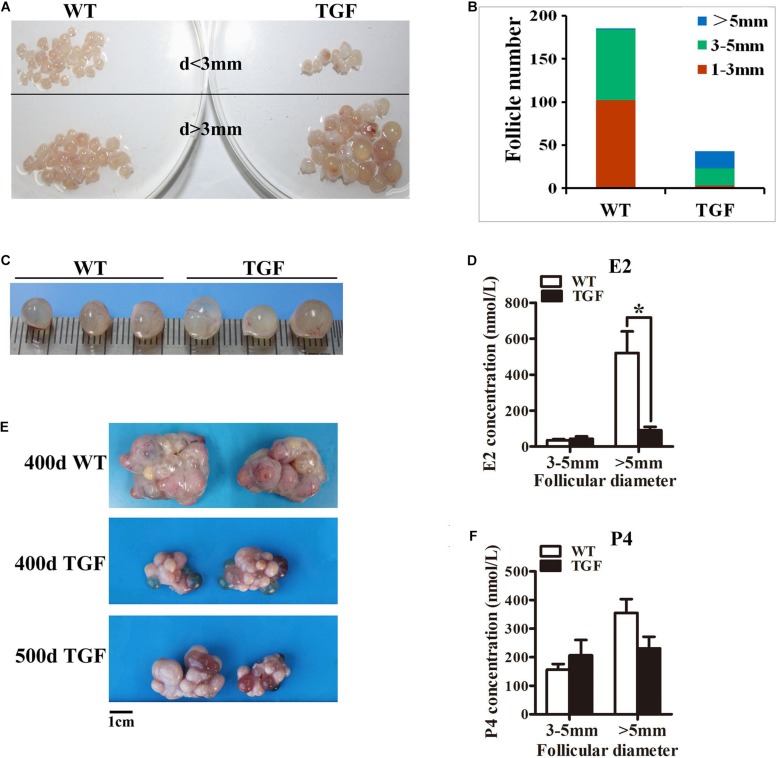
Transgenic ovaries contained abnormally enlarged antral follicles with dramatically reduced concentration of follicular fluid E2, resulting in delayed ovulation. **(A)** Photograms of the isolated antral follicle of 365-day ovaries demonstrated remarkably declined number of antral follicles with a diameter <3 mm in TGF ovaries. **(B)** Statistical data showed reduced total number of antral follicles, and substantially increased number of follicles with a diameter >5 mm in 365-day TGF ovaries. Antral follicles were isolated from three ovaries of different gilts, and then classified into three groups according to their diameter (*d* 1–3 mm, *d* 3–5 mm, and *d* >5 mm). **(C)** Comparison of the three largest follicles isolated from WT and TGF ovaries. **(D)** E2 concentration in follicular fluid of TGF large antral follicles was significantly lower than that in WT pre-ovulatory follicles. ^∗^ *p* < 0.05. **(E)** Many corpus lutea were observed on the surface of both 400 and 500-day old TGF ovaries, but the ovaries were apparently smaller than 400-day old WT ovaries. **(F)** P4 concentrations in the follicular fluids of TGF and WT were not significantly different. The hormones in follicular fluid were measured in follicles of three 365-day old ovaries of different gilts.

Because the estrous cycle is determined by ovarian and follicular development (Foxcroft and Hunter, 1985; Noguchi et al., 2010), the disordered estrous cycle of TG gilts is potentially caused by impaired ovarian follicular development. In this regard, ovaries from gilts of different ages were collected and processed for morphological examination. Surprisingly, we found remarkably decreased size in TG ovarian and number of AFs on the surface of TG ovaries of 140–365-day old gilts. In addition, apparent size difference was observed between bilateral TG ovaries (Figure 3A). Corpus lutein was not observed in TG ovaries from 140–365-day old gilts, but can be found in 400- and 500-day TGF ovaries (Figure 4E). Besides, the weight of TG ovaries before sexual maturity was markedly lower than that of WT ovaries (Supplementary Table S4). Among the TG ovaries, we discovered eight streak ovaries in six gilts, denoted as TGS ovaries with an incidence of about 14%, whereas no streak ovary was found in WT ovaries (Figures 3A,B). These TGS ovaries contained none or less than three visible AFs on the ovarian surface. In the cortex of TGS ovaries from 110- and 200-day old gilts, most follicles were arrested in the primary stage (Figures 3B,E). In the cortex of TGS ovaries from 365-day old gilts, most follicles were arrested in the secondary stage, and the degradation of follicles became apparent (Figure 3B). Unlike TGS ovaries, the rest of TG ovaries contained many visible large AFs on the surface, and we denoted them as TGF ovaries (Figures 3A,C). Different stage of follicles can be found in these TGF ovaries; however, the follicle number decreased drastically during follicular development (Figures 3C–E). Notably, during the early follicle stage, the significant decline in primordial and PF number led to a much thinner ovarian cortex in the TGF ovaries of pre-puberty gilts. In addition, structural abnormality of SFs was evident, particularly in the TGF ovaries of gilts undergoing puberty (Figures 3C,G,H and Supplementary Figure S3A). We observed abnormally enlarged (Figure 3H) or degenerated oocytes (Figure 3Gii), multioocytic follicles (Figure 3Gi), highly irregular GC layers (Figures 3Gii,iv), degraded GCs (Figure 3Giv), and abnormally thickened theca layers (Figure 3Giii). Furthermore, ovarian sections from five TG gilts at the age of 160–400 days showed a markedly reduced proportion of normal SFs in the TGF ovaries (Figure 3F and Supplementary Figure S3A).

Histological observation also revealed some striking features of TGF AFs. Most notably, AF number declined remarkably, but its antrum was enlarged substantially (Figure 3C) and surrounded with loosely organized smaller GCs (Figure 3I). We further isolated AFs from three 365-day TGF ovaries derived from different gilts for statistical analysis. The results showed that TGF ovaries contained less total number of AFs and less number of small AFs (diameter <5 mm); however, it contained substantially more large AFs (diameter >5 mm) (Figures 4A,B). We found that the diameter of the largest AF in TGF ovary can be up to 9 mm, whereas it is about 7 mm in WT ovaries (Figure 4C). Normally, porcine AFs stop growth at a diameter of about 5 mm, and only selected follicles continue to grow through the accumulation of follicular fluid and ovulate at a diameter of about 7 mm (Soede et al., 2011). Thus, the increased numbers of abnormally enlarged AFs in the TGF ovaries may be related to dysovulation and the disordered serum reproductive hormones found in TG gilts. Subsequent measurement of the concentration of reproductive hormones in follicular fluid of TGF large AFs (diameter >5 mm) showed that the E2 concentration was remarkably lower (Figure 4D), but the concentrations of the other three hormones including P4 (Figure 4F), FSH (Supplementary Figure S3B), and LH (Supplementary Figure S3C) was not significantly different from those in WT large AFs. Reduced E2 production in the TGF large follicles may imply an absence of dominant follicle selection (Clement and Monniaux, 2013). Taken together, these results provide convincing evidence that the knockdown of *BMP15* could severely inhibit both follicular development and ovulation of poly-ovulatory pig.

### Knockdown of *BMP15* Caused Premature Luteinization and Impaired Oocyte Quality in TGF Follicles

**FIGURE 5:**
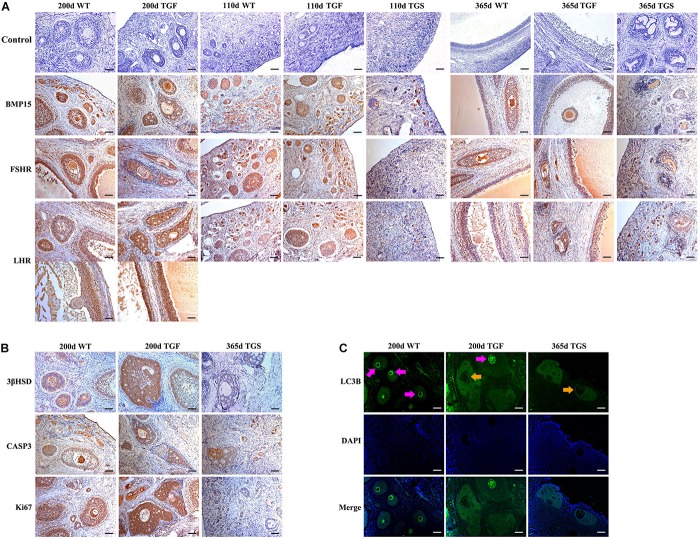
Abnormal TGF follicles showed premature luteinization and impaired oocyte quality. **(A)** Immunostaining of ovarian sections indicated that the expression of BMP15 decreased remarkably in TGS abnormal follicles, but was only slightly reduced in TGF follicles, compared with WT follicles. FSHR shared a similar expression pattern with BMP15, and the expression pattern of LHR was different from that of BMP15. It expressed higher in TGF GCs of both preantral and antral follicles. Scale bar = 100 μm. **(B)** Follicular cell apoptosis, proliferation, and premature luteinization were evaluated by immunostaining with Caspase3, Ki67, and 3βHSD, respectively. Notably, higher expression level of 3βHSD was discovered in abnormal TGF follicles. However, the expression of Caspase3 and Ki67 in abnormal TGF follicles was not significantly different from that in WT follicles. Scale bar = 100 μm. **(C)** Immunofluorescence images demonstrates intensive expression of autophagy-related protein, LC3B, in oocytes of normal follicles of TGF and WT ovaries, but it was barely expressed in oocytes of abnormal follicles of TG (TGF and TGS) ovaries. Purple arrow, oocytes in normal follicles; Orange arrow, oocytes in abnormal follicles. Scale bar = 100 μm.

We examined the expression and activation of factors associated with follicular development. The results confirmed that BMP15 protein is abundantly located in both WT oocytes and GCs of primary to pre-ovulatory follicles. Both normal and abnormal TGF follicles showed slightly decreased BMP15 protein accumulation in the less degraded GCs than in WT. However, markedly reduced BMP15 expression level was noted in deteriorated oocytes of TGF abnormal (TGFA) follicles. TGS ovaries exhibited the minimum BMP15 protein level in the PFs of 110-day TGS ovaries and highly degraded SFs of 365-day TGS ovaries (Figure 5A and Supplementary Table S5). Thus, we speculated that the *in vivo BMP15* interference efficiency is different in TG individuals, which possibly explains the two TG ovarian phenotypes (TGF and TGS). Besides, TGS ovaries displayed a phenotype of high degradation and serious inhibition of follicular development and cellular activity in the arrested SFs, similar to the phenotypes of BMP15 homozygotes mutations in sheep (Braw-Tal et al., 1993) and in women with POI (Luisi et al., 2015). Hence, we focused on the effects of TGF follicles.

**FIGURE 6:**
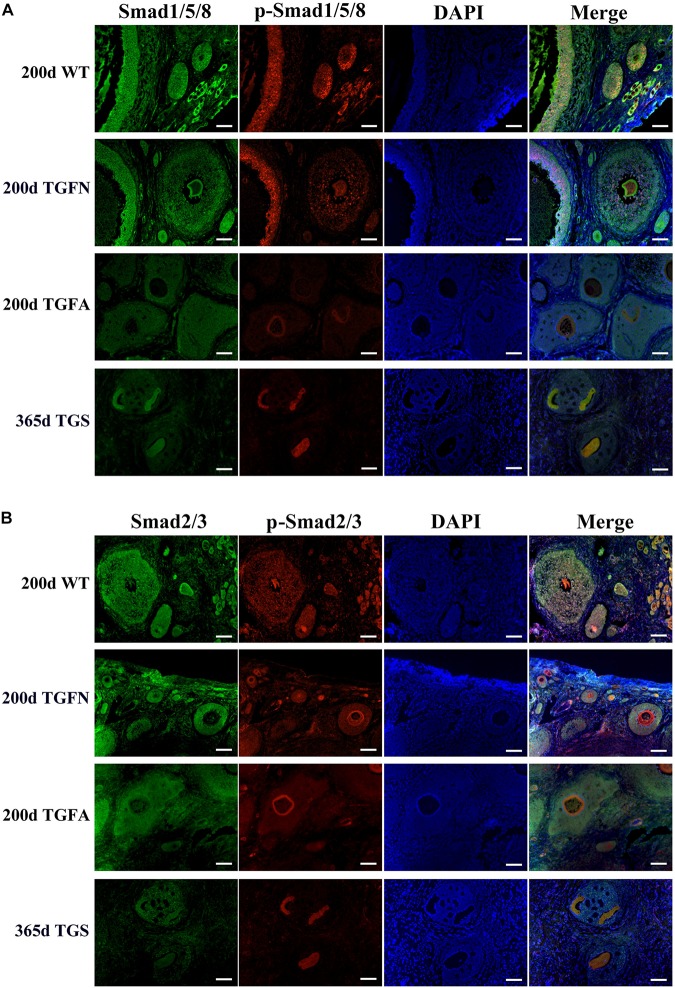
Smad1/5/8 signaling pathway was inhibited in abnormal follicles of TGF ovaries. **(A)** Immunofluorescence images showed Smad1/5/8 pathway was evidently less activated in abnormal follicles of TGF ovaries, and severely inhibited in highly degraded 365-day old TGS follicles, compared with normal follicle of TGF and WT ovaries. **(B)** Immunofluorescence images demonstrated a mild decrease in Smad2/3 signaling in both normal and abnormal follicles of TG ovaries. Smad2/3 signaling was remarkably inhibited in highly degraded 365-day TGS follicles. TGFN, normal follicles in TGF ovary; TGFA, abnormal follicles in TGF ovary. Scale bar = 100 μm.

In TGF follicles, we found that the expression patterns of both GDF9 and FSHR, the cooperator and down regulator of BMP15, respectively, corresponded with that of BMP15 (Figure 5A, Supplementary Figure S4, and Supplementary Table S5) in TGF follicles. However, there were no changes in the expression of BMP15 receptors (ALK6 and BMPR2) (Supplementary Figure S4). Unlike BMP15, the expression of LHR was higher in TGF follicles than in WT (Figure 5A). The excess expression of LHR suggested premature luteinization in TGF follicles, which was also demonstrated by the significantly raised expression of 3β-hydroxysteroid dehydrogenase (Grasa et al., 2016) in TGF SFs. In consideration of the striking features of reduced follicle number and degraded GCs in the TGF follicles, we determined the expression levels of caspase 3 and Ki67 to assess cell apoptosis and proliferation activity. Surprisingly, there was no change in the expression of both caspase 3 and Ki67 in the degraded TGFA follicles (Figure 5B). Subsequent investigation of BMP15-mediated signaling pathways suggested an underlying mechanism. We observed notably weakened Smad1/5/8 activity in TGFA follicles when compared to TGF normal SFs (Figure 6A and Supplementary Figure S5), but slighter attenuated Smad2/3 phosphorylation was shown in the abnormal follicles (Figure 6B and Supplementary Figure S5). It is likely that Smad1/5/8 mainly contributed to the inhibition of follicular development in the TGFA follicles, and Smad2/3 activation by the BMP15-independent pathway played a role in the growth of the less degraded follicles. Except for GCs, we also discovered impaired oocyte quality in TGFA follicles. As shown in Figure 5C, an undetectable level of autophagy-related protein LC3B (microtubule-associated protein 1 light chain 3) (Jiang et al., 2017) was observed in the oocytes of TGFA SFs, whereas the oocytes of WT and TGF normal follicles displayed a normal level of LC3B. This result demonstrates that autophagy activity of the oocytes of TGFA follicles, which is fundamental to the cellular processes of the oocytes, was largely weakened (Su et al., 2017).

### Knockdown of *BMP15* Resulted in Dynamic Transcriptomic Alteration During TGF Follicular Growth

**FIGURE 7:**
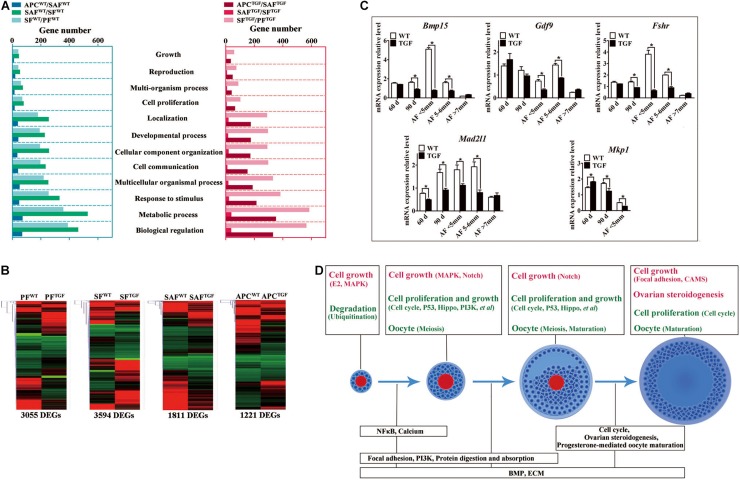
Altered follicle dynamic transcriptomes during TGF follicular development. **(A)** Different numbers of DEGs were presented in enriched 12 biological processes (GO) of WT and TGF dynamic transcriptomes during follicular development. In TGF ovary, the least DEGs was presented in enriched GO during the transition from secondary to small antrum follicle stage; in WT ovary, the least number of DEGs was presented in enriched GO during the transition from small antrum to antral follicle. The highest number of DEGs was presented in enriched GO during the transition from primary to secondary follicle stage in TGF ovary, whereas in WT ovary, this was presented during the transition from secondary to small antrum follicle stage. Gene ontology-based analysis was conducted with Webgestalt software. **(B)** Both DEGs number and their clustering pattern revealed a significant difference between the gene expression of WT and TGF follicle during each follicle stage. **(C)** A subset of DEGs was validated by qPCR. 60 *d* stands for 60-day ovarian tissue, which was mainly composed of early stage follicles (primordial, primary, and early secondary follicles). 90 *d* represents 90-day ovarian tissue, which was mainly composed of secondary follicles without antral follicles. AF <5 mm, antral follicle with a diameter <5 mm. AF 5–6 mm, antral follicle with a diameter of 5–6 mm. AF >7 mm, antral follicle with diameter >7 mm. **(D)** Summary of important pathways (based on inter-effect analysis) involved in follicular development. Up-regulated pathways are shown in red, and down-regulated pathways are shown in green. ^∗^Stands for statistical significance (*P* < 0.05).

To further investigate the regulatory role of BMP15 in porcine follicular development, RNA-seq was carried out on follicles or GCs captured by LCM method from frozen sections of both WT and TGF ovaries (Fend et al., 1999; Bonnet et al., 2015). LCM-captured follicles were categorized into three stages of follicular development: PF, SF, and SAF stages. For large antrum follicle, only parietal granulosa and theca cells (APC) were captured by LCM for RNA-seq. The follicles or APCs were captured from the frozen sections of each of the five TGF and WT ovaries of gilts at age ranging from 60–170 days. The gene profiles of 34,640 genes generated by RNA-seq were used for the identification of DEGs, as well as GO and pathway enrichment analysis basing on intra- (between each two continuous follicle stages in either WT or TGF sample) and inter- (between each follicle stage of WT and TGF sample) effect comparisons (Table 1). In intra-effect comparisons, the largest number of DEGs (3503 DEGs) was found in SAF^*WT*^/SF^*WT*^ comparison, and the least number of DEGs (350 DEGs) was found in APC^*WT*^/SAF^*WT*^ comparison. However, in contrast to WT, during TGF follicular development, the lowest number of DEGs was found in SAF^*TGF*^/SF^*TGF*^ comparison, and the largest number of DEGs was found in SF^*TGF*^/PF^*TGF*^ comparison. A striking difference in the dynamics of transcriptions between WT and TGF follicular development was also found in GO (Figure 7A) and pathway (Supplementary Figure S6A) enrichment. In WT ovary, more DEGs were presented in the enriched GO during the dynamical transition from early primary to SF stage, and from secondary to SAF stage, but less DEGs were presented in enriched GO during SAF to large antrum follicle stage transition. However, in TGF ovary, more DEGs were presented in enriched GO during the dynamical transition from SAF to large antrum follicle stage, but less DEGs were presented during secondary to SAF stage transition. These results in GO enrichment were consistent with that found in pathway enrichment. Based on the intra-effect analysis, GCs differentiation during the late secondary stage and early antrum formation may have been delayed during TGF follicular dynamical development (Hennet and Combelles, 2012). BMP15 probably played a more important role during the dynamic development of secondary and subsequent follicle stages than in the early stages.

**TABLE 1:** Summary of DEGs in comparisons.

**Comparisons**	**Total**	**Up-regulated**	**Down-regulated**
**Intra effect**			
SF^*WT*^/PF^*WT*^	2877	1099	1778
SAF^*WT*^/SF^*WT*^	3503	1168	2335
APC^*WT*^/SAF^*WT*^	350	163	187
SF^*TGF*^/PF^*TGF*^	4503	1594	2909
SAF^*TGF*^/SF^*TGF*^	236	74	162
APC^*TGF*^/SAF^*TGF*^	2390	1126	1264
**Inter effect**			
PF^*TGF*^/PF^*WT*^	3055	1482	1573
SF^*TGF*^/SF^*WT*^	3594	1658	1936
SAF^*TGF*^/SAF^*WT*^	1812	914	898
APC^*TGF*^/APC^*WT*^	1221	606	615

*PF, primary follicle; SF, secondary follicle; SAF, small antrum follicle; APC, large antrum parietal cell.*

Considering that the expression and function of BMP15 during follicular development are stage-specific (Paradis et al., 2009; Sun et al., 2010), we conducted the RNA-seq data analysis based on inter-effect comparisons. Both DEGs number and their clustering results revealed distinct effect on the knock down of BMP15 in each follicle stage, where the largest number of DEGs was found in the SF stage (Figure 7B). Based on these DEGs, we enriched a total of 15 up-regulated and 26 down-regulated pathways (Supplementary Figure S6B) in all the four follicle stages. Ten pathways were enriched during the three dynamical transitions of each of the two continuous follicle stages, in which DEGs were identified based on the combination of the intra- and inter-effect comparison (Supplementary Figure S7B). These pathways are illustrated in Figure 7D according to their relevant function. Interestingly, the knock down of BMP15 seemed to lead to an increased cell growth in PF, due to the significant up-regulation of estrogen and MAPK pathways and the down-regulation of ubiquitin protein degradation pathway. However, knocking down BMP15 likely inhibited GCs proliferation and growth from the SF stage onward, because of the significant down-regulation of pathways such as cell cycle, Hippo, P53, and PI3K, which also implies the potential involvement of BMP15 in these pathways. Though GCs proliferation and growth were inhibited by knocking down BMP15, the significantly up-regulated MAPK pathways in primary and SF stages, and Notch pathway in secondary and SAF stages may compensate for the knock down by promoting cell proliferation and growth in preantral follicles to support the continuous development of certain number of follicles to maturity. Furthermore, knocking down BMP15 did not result in significant pathway alteration during the transition from secondary to SAF stage (Figure 7D). In addition to the fact that the minimum DEGs was presented in SAF^*TGF*^/SF^*TGF*^ comparison (Table 1), our findings also suggest that knocking down BMP15 may cause an inhibition of GCs differentiation and abnormal development in preantral follicles. In the large antrum stage, DEGs involved in ovarian steroidogenesis (*Lhr*, *Cyp17, 3*β*Hsd, etc.*) were significantly up-regulated in theca cells of TGF follicles (Supplementary Table S6), possibly associated with premature luteinization of TGF follicles (Figure 5B). Except for GCs, we also enriched significantly down-regulated DEGs involved in oocyte meiosis and maturation in TGF follicles beyond the PF stage, which was possibly related to the impaired oocyte quality (Figure 5C).

Moreover, we found that *in vivo* knock down of BMP15 resulted in significant decrease in *Bmp15* expression level from primary to SAF stage (Supplementary Table S6), which was confirmed by qPCR analysis (Figure 7C). Through a correlation analysis of the DEGs, we predicted 13 downstream regulated genes of *Bmp15*, in which six genes (*Atrx*, *Amd1*, *Dtd2*, etc.) were positively correlated, and seven genes (*Fgf9*, *Igfbp7*, *Cmpk2*, etc.) were negatively correlated (Supplementary Figure S7A and Supplementary Table S7). Furthermore, an unexpected significantly decreased expression of *Fshr* in TGF follicles was detected by transcriptomic analysis (Supplementary Table S6), and confirmed by qPCR (Figure 7C), which seemed to be inconsistent with the previous perspective that BMP15 played a role in the suppression of *Fshr* expression (Otsuka et al., 2001; McMahon et al., 2008; Abir and Fisch, 2011; Shimizu et al., 2019). This discrepancy was probably due to species difference or the abnormal development of TGF GCs, including degradation and premature luteinization. In addition, mRNA level of mitotic spindle assembly checkpoint protein (*Mad2l1*) decreased significantly in TGF ovarian tissues and antrum follicles, implying the inhibition of TGF cell mitosis. Increased expression of *Mkp1* (*Dual specificity protein phosphatase 1*) in 60-day old TGF ovarian tissues but decreased expression of this gene in 60-day TGF ovarian tissues and antrum follicles (Figure 7C) seemed to be related to the up-regulated MAPK pathway in early TGF preantral follicles (Figure 7D).

### Knockdown of *BMP15* Caused Reduced Capacity of TGF Follicles to Ovulate

**FIGURE 8:**
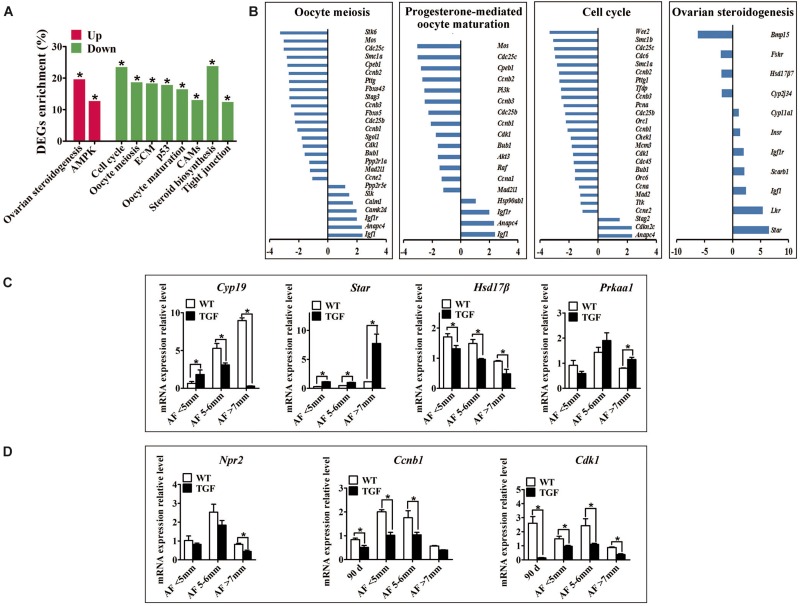
Single cell RNA-seq on TGF COCs showed affected process in ovulation. **(A)** Pathway enrichment revealed two significantly up-regulated pathways which potentially contributed to increased number of large antral follicles, and eight significantly down-regulated pathways that are involved in cumulus cell function and oocyte maturation. **(B)** Pathways in oocyte meiosis, progesterone-mediated oocyte maturation, ovarian steroidogenesis, and cell cycle, which are closely related to oocyte maturation and ovulation, showed DEGs enrichment of 22.4, 21.2, 25.5, and 22.6%, respectively. **(C)** qPCR validation of mRNA expression level of DEGs associated with the pathways in ovarian steroidogenesis (*Cyp19*, *Star*, *Hsd17*β) and AMPK (*Prkaa1*) pathway. **(D)** Quantification of mRNA level of genes related to oocyte meiosis indicated decreased expression of MPF, but unaffected expression of *Npr2* during TGF follicular development. 90 *d* represents 90-day ovarian tissues. ^∗^Stands for statistical significance (*P* < 0.05).

Evidence of disordered estrous cycle, abnormally enlarged AFs, and no corpus lutein observed in sexually mature TG gilts until 365-day old demonstrate that knockdown of *Bmp15* could cause dysovulation. To investigate the underlying factors causing dysovulation, COCs were isolated from antrum follicles with a diameter of 5–7 mm for single-cell RNA sequencing. As expected, sequencing results showed a drastic reduction in *BMP15* in TGF COCs (Table 2), which was confirmed by qPCR analysis in antrum follicles (Figure 7C). Interestingly, GDF9, the closely related homologous protein of BMP15, was down-regulated with the knock down of BMP15 (Figure 7C and Table 2). However, the expression of other BMP proteins (BMP4 and BMP6) was not affected by the knock down of BMP15. Surprisingly, BMP15 receptors (*Bmpr2* and *Alk6*) as well as its signaling protein, *Smad8*, were significantly up-regulated, which was probably related to the increased expression of *Fst* and *Inh*α or another activin. About four-folds decreased expression of both *Fshr* and *Hsd17*β (Luu-The et al., 2006; Figure 8C and Table 2) might have contributed to the significantly reduced E2 production and the absence of dominant follicle selection in TGF antrum follicles. The up-regulated expression of steroidogenesis related factors including *Lhr*, *Star*, *Cyp11a* (*cytochrome P450 family 11 subfamily A member*), *Cyp19a* (*cytochrome P450 family 19 subfamily A member*) (*steroidogenic acute regulatory protein*) (Figures 8B,C and Table 2) in TGF COCs was consistent with the results of transcriptomic analysis of TGF APC (Supplementary Table S6), which might have contributed to premature luteinization (Jakimiuk et al., 2001; Stocco, 2001; Magoffin, 2005; Long et al., 2009). It has been reported that the down-regulated expression of *Amhr2* (Anti-Mullerian hormone receptor type 2) and *Cx43* (Gap junction protein alpha 1) induced by LH in preovulatory follicles was important to ovulation (Norris et al., 2008; Pierre et al., 2013). Thus, the increased expression of these two genes in TGF COCs potentially resulted in a decreased capacity of oocyte meiosis resumption and ovulation. In addition, markedly decreased expression of oocyte quality related genes (*Bmp15*, *Gdf9*, *Zp2*, *Zp3*, *Zar1*, and *Irf6*) strongly implies a reduced oocyte competence in TGF COCs (Picton et al., 1998; Wu et al., 2003, 2007; Hussein et al., 2006; Sabel et al., 2009).

**TABLE 2:** Interest genes that related to COCs function.

**Genes**	**Log2 fold change**	***p*-value**	**Description**
**BMPs and receptors**			
*BMP15*	−6.21	<0.01	Bone morphogenetic protein 15
*Gdf9*	−3.38	<0.01	Growth differentiation factor 9
*Bmp6*		ns	Bone morphogenetic protein 6
*Bmp4*		ns	Bone morphogenetic protein 4
*Bmpr2*	2.37	<0.01	Bone morphogenetic protein receptor type II
*Alk6*	1.52	<0.01	Bone morphogenetic protein receptor type-IB
**Hormones and receptors**			
*Fshr*	−2.14	<0.01	Follicle-stimulating hormone receptor
*Fst*	1.12	<0.01	Follistatin
*Pgrmc1*	1.27	<0.01	Progesterone receptor membrane component 1
*Pgrmc2*	1.86	<0.01	Progesterone receptor membrane component 2
*Inhα*	2.07	<0.01	Inhibin alpha
*Amhr2*	3.94	<0.01	Anti-Mullerian hormone receptor type 2
*Lhr*	5.40	<0.01	Luteinizing hormone/choriogonadotropin receptor
*Esr1*	1.55	<0.01	Estrogen receptor 1
*Esr2*		ns	Estrogen receptor 2
**Steroidogenesis related factors**			
*Star*	6.52	<0.01	Steroidogenic acute regulatory protein
*Hsd17β7*	−2.0	<0.01	Hydroxysteroid 17-beta dehydrogenase 7
*Cyp11a*	1.19	<0.01	Cytochrome P450 family 11 subfamily A
*Cyp19a*	5.45	<0.01	Cytochrome P450 19A2
**Oocyte quality related factors**			
*Zp2*	−2.81	<0.01	Zona pellucida sperm-binding protein 2 precursor
*Zp3*	−2.97	<0.01	Zona pellucida sperm-binding protein 3
*Zar1*	−2.88	<0.01	zygote arrest 1
*Irf6*	−1.7	<0.01	Interferon regulatory factor 6
**Others**			
*Smad8*	3.50	<0.01	SMAD family member 8
*Igf1*	2.40	<0.01	Insulin-like growth factor I
*Cx43*	2.86	<0.01	Gap junction protein alpha 1

A total of 2,820 DEGs (885 up-regulated, 1,935 down-regulated) was generated for pathway enrichment. The significantly up-regulated AMPK and ovarian steroidogenesis (Figures 8A–C) pathways were likely to contribute to the greater number of large antrum follicles in TGF ovaries, according to the findings of previous studies in sheep (Foroughinia et al., 2017) and sow (Knox, 2005). However, pathways (e.g., Cell cycle, P53) involved in cell proliferation and growth were significantly down-regulated (Figures 8A,B), which was consistent with the results of the dynamic transcriptomic analysis of TGF follicles (Supplementary Figure S6B). Furthermore, four pathways including oocyte meiosis, oocyte maturation, cell cycle, and ovarian steroidogenesis, which were closely involved in the regulation of oocyte maturation and ovulation, presented DEGs enrichment of more than 20% (Figures 8A,B). In total, these results revealed both impaired function of cumulus cells and oocyte maturation in TGF COCs, suggesting a reduced capacity to ovulate.

However, surprisingly, the expression of *inosine monophosphate dehydrogenase 2* (*Impdh*) and *natriuretic peptide receptor 2* (*Npr2*) was not affected. These two genes have been reported in mice to be up-regulated by BMP15 and GDF9 during the activation of maturation promoting factor (MPF) [Cyclin B and Cyclin dependent kinase 1 (CDK1)] and stimulation of oocyte meiotic resumption *in vitro* (Wigglesworth et al., 2013). Instead, we discovered significantly decreased expression of *Cyclin B* and *Cdk1* in TGF follicles from secondary stage onward (Figure 8D and Supplementary Table S8), which implies the involvement of BMP15 in the modulation of porcine oocyte meiosis possibly by regulating the expression of MPF.

## Discussion

The effect of BMP15 mutations on ovarian follicular development and ovulation rate was first discovered in Inverdale (FecX) sheep (Davis et al., 1992; Braw-Tal et al., 1993; Smith et al., 1997). In these sheep, ewes with single allele of inactive *BMP15* gene showed increased ovulation rate and a higher incidence of twin or triplet births, whereas ewes with bi-alleles of inactive *BMP15* gene were sterile with primary ovarian failure phenotype (Galloway et al., 2000). Studies on animals immunized with different regions of BMP15 peptide revealed that an increased ovulation rate can be found in females where some of the BMP15 have been neutralized, but an inhibition of follicular growth and ovulation was found in females where most of the active BMP15 has been neutralized (McNatty et al., 2007). Therefore, different extent of reduction of biologically active BMP15 protein level seems to have different influence on fertility. In this study, we found two different ovarian phenotypes (TGS and TGF) in our *BMP15* knockdown gilts. The different ovarian phenotypes might be caused by different *in vivo* expression level of BMP15. Indeed, in TGS ovaries, the marked reduced level of both mRNA (Figure 1E) and protein (Figure 5A) of BMP15 revealed that the majority of BMP15 was knocked down by integrated shRNA. In contrast, BMP15 protein accumulated abundantly in TGF ovaries, and was only slightly lower than that in WT ovaries, despite the fact that the mRNA level of BMP15 had decreased to half of that of the wild-type as detected in 365-day (Figure 1E) and 30-day (Figures 1F,G) old ovarian tissues. Therefore, the low interference of BMP15 expression in TGF ovaries may confer on them a less severely impaired ovarian phenotype, as TGF ovaries contained each stage of follicle but presented remarkably reduced follicle number, increased ratio of abnormal follicle, disordered reproductive hormones, and ovulation dysfunction. We found that TGF and TGS ovaries could be concurrently present in a single TG gilt (Figure 3A), and this may be caused by epigenetic mechanisms including DNA methylation and histone modifications (Spencer et al., 2015), which could cause dynamic and heterogeneous expression of shRNA from integrated TG construct in different individuals and even in different ovaries within a single individual, explaining the varied *in vivo* levels of active BMP15 in each ovary of each individual. We suspect that in some critical stages of early follicle development, there may be a threshold level of active BMP15 that supports the development of follicles into subsequent stages, indicating that the lack of BMP15 in a specific ovary may hamper the transition of the follicles into the subsequent developmental stages. Previous reports in sheep revealed that heterozygous mutations in BMP15 inhibit GCs growth but increase GCs sensitivity to FSH, leading to increased ovulation in smaller matured follicles with reduced amounts of E2 and inhibin (Shackell et al., 1993; Otsuka et al., 2000, 2001; Fabre et al., 2006). However, this was inconsistent with our results. The *in vivo* mRNA level of BMP15 in TGF ovaries was knocked down to half of that in wild-type, and thus, TG gilts with TGF ovaries could be considered as pigs with heterozygous mutations in BMP15. In contrast to ewes heterozygous for mutations in BMP15, our TG gilts presented impaired ovulation, as corpus lutein could not be found in TGF ovaries from TG gilts younger than 365 days, though they can be observed in TGF ovaries from 400- and 500-day old TGF gilts (Figure 4E), indicating a delayed ovulation in TGF gilts. In addition, significantly decreased amount of total and smaller AFs in TGF ovaries (Figures 4A,B) seemed incapable of supporting an increased ovulation rate. Moreover, the appearance of abnormally enlarged AFs with lower FSHR expression level and E2 production (Figure 4D) may indicate that TGF follicles could not ovulate at normal size probably due to the attenuated FSH sensitivity and a lack of dominant follicle selection in TG gilts. Besides, premature luteinization of GCs possibly caused by insufficient FSH stimulation may also contribute to the dysovulation of TGF gilts. Thus, the results in TGF puberty gilts were different from that of the poly-ovulatory *Bmp15^–/–^* mice, which showed normal follicular development and could ovulate at puberty (Yan et al., 2001). Hence, we suggest that the role of BMP15 in ovulation is species-specific and different not only between mono- and poly-ovulatory mammals, but also between poly-ovulatory species.

Bone morphogenetic protein 15 suppresses *Fshr* expression in GCs, which affects GCs proliferation and steroidogenesis in the AFs of rodents (Otsuka et al., 2001; McMahon et al., 2008) and humans (Abir and Fisch, 2011; Shimizu et al., 2019). Previous studies on sheep indicated that heterozygous mutations in BMP15 could increase the sensitivity of GCs to FSH stimulation in the AF, leading to increased ovulation rate (Fabre et al., 2006). However, a recent study showed that treatment with BMP15 caused increased expression of *Fshr* in bovine preantral follicles after 12 days of culture (Passos et al., 2013). These conflicting reports are probably caused by species-specific differences and the different response to BMP15 stimulation in each follicular development stages. In this study, we found that, in contrast to the results found in sheep, the knockdown of *BMP15* did not increase but significantly inhibited *Fshr* expression in both preantral and AFs (Figure 7C, Table 2 and Supplementary Table S6). Furthermore, the inhibition in GCs proliferation and differentiation, increased expression of genes involved in steroidogenesis (*StAR*, *Cyp11a*, and 3βHSD) (Figures 5B, 8C, Table 2 and Supplementary Table S6), drastic reduction of E2 production (Figure 4E), and subsequent absence of dominant follicle selection in the TGF follicles, were likely the consequences of the declined sensitivity of GCs to FSH. Suppression of *FSHR* expression in preantral follicles of TG gilts implies that BMP15 could stimulate porcine follicle growth and development at an earlier follicle stage rather than at gonadotropin-dependent period (Mori, 2016). Given the degradation of GCs and abnormal structure of GCs layers observed in TGF ovaries, another possible reason for the decreased expression of FSHR might be related to the impaired development of GCs caused by BMP15 deficiency.

As a paracrine and autocrine factor of oocyte, BMP15 can promote not only the development of follicular somatic cells, but also the development of oocyte itself. Studies on the *in vitro* maturation of oocyte have demonstrated that BMP15 are able to stimulate cumulus cell expansion (Braw-Tal et al., 1993; Sugiura et al., 2010; Peng et al., 2013; Lin et al., 2014; Sudiman et al., 2014), promote the signaling of LH-induced maturation of the cumulus-oocyte complex (Su et al., 2010), improve oocyte quality (Hussein et al., 2006; Caixeta et al., 2013), and increase blastocyst rate and embryonic development of fertilized oocytes (Wu et al., 2007; Gode et al., 2011). Recently, the expression level of BMP15 has been suggested as a diagnostic marker of oocyte quality (Wu et al., 2007). In this study, we confirmed the important role of BMP15 in porcine oocyte development *in vivo*. We found that the knockdown of BMP15 could cause oocyte degeneration or abnormal enlargement (Figures 3G,H), and lack of normal autophagy activity in oocytes of abnormal preantral follicles (Figure 5C). Further transcriptomic analysis of follicle and COCs also implies that genes and pathways involved in oocyte meiosis and maturation in TGF follicles were affected from secondary stage onward (Figures 7D, 8A). Previous studies have indicated the possible mechanisms of action of BMP15 in the regulation of oocyte meiosis. One study reported that BMP15 and GDF9 can promote oocyte meiotic resumption in mice through the up-regulation of *Npr2* and *Impdh* (Wigglesworth et al., 2013). Another study showed that inhibiting BMP15 signaling pathway by Smad2/3 phosphorylation inhibitor resulted in significantly decreased expression of *Cdc2* and *Cyclinb1* during porcine oocyte *in vitro* maturation (Lin et al., 2014). However, our transcriptomic results showed that the expression level of *Npr2* and *Impdh* were not affected in both TGF follicles and COCs, instead, the expression levels of MPF (*Cdc2* and *Cyclinb1*) decreased significantly (Figure 8D and Supplementary Table S8). Therefore, our findings demonstrate that the regulation of MPF expression is associated with the role of BMP15 in porcine oocyte meiosis and maturation; however, this requires further elucidation.

**FIGURE 9:**
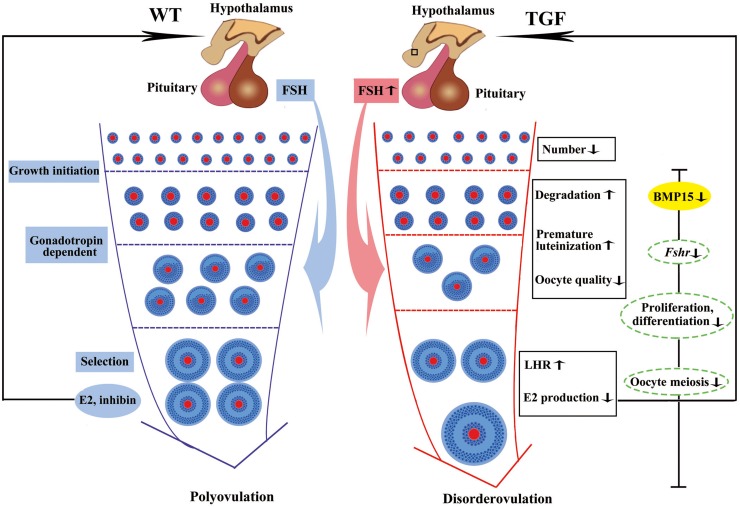
Summary of biological functions and possible regulatory mechanism of BMP15 in TGF follicular development. Compared to WT gilt, knockdown of BMP15 caused remarkable reduction of follicle number during the TGF follicular developmental process, accompanied by impaired oocyte quality, degradation, and premature luteinization in TGF preantral follicles. This may lead to increased expression of LHR and dramatically decreased E2 production in TGF antral follicles, resulting in lack of dominant follicle selection but abnormally enlarged antral follicles, presenting a dysovulation phenotype. Decreased E2 production in TGF antral follicles may have a negative feedback to pituitary to increase the expression of *Fsh*. However, the decreased expression of *Fsh* receptor, *Fshr*, in GCs beyond the PF stage, by knocking down BMP15, attenuates the stimulation of antral follicles growth. In addition, BMP15 knockdown leads to the inhibition of cell proliferation and differentiation, declined oocyte quality, and meiotic maturation during TGF follicular development, contributing to the appearance of enlarged follicle in TGF ovary and dysovulation. Colored ellipses represent results based on morphological observation and molecular examination. Dotted ellipses represent results based on transcriptomic analysis.

In summary, the knockdown of *BMP15* caused markedly reduced fertility of TG gilts mainly through the inhibition of both GCs and oocyte development (Figure 9). The suppression of GCs proliferation and differentiation led to decline in the number of early follicles, GCs degradation, and reduced sensitivity of GCs to FSH stimulation, consequently leading to premature luteinization, higher LHR expression, and lower E2 production in large AFs. The effect on oocyte development directly led to impaired oocyte quality and oocyte meiotic maturation. Consequently, large AFs become abnormally enlarged, resulting in dysovulation and disordered reproductive hormones. Our results revealed a remarkable physiological suppression of porcine ovarian follicular development and ovulation in *BMP15* knockdown gilts, demonstrating an essential role of BMP15 on porcine female reproduction and providing new insights into the regulatory role of BMP15 in poly-ovulatory mammals. Our findings provide the basis for further investigation on the complicated regulatory function of BMP15 in female fertility of poly-ovulatory species and the development of possible strategies for improving porcine female fertility through the modulation of BMP15 expression.

## Data Availability Statement

The authors confirm that all data underlying the findings are fully available without restriction. Data are available from the Sequence Read Archive (SRA) under Accession no. PRJNA561679.

## Ethics Statement

All procedures were performed in strict accordance with the recommendations of the Guide for the Care and Use of Laboratory Animals of the National Institutes of Health. The protocol was approved by the Institutional Animal Care and Use Committee (IACUC), Sun Yat-sen University (approval Number: IACUC-DD-16-0901).

## Author Contributions

ZH, YC, and XHL conceived and supervised the project. YQ, ZL, XY, and ZH contributed to the transgenic gilts generation. YQ, TT, WL, XS, GS, XFL, MW, and XYL contributed to the tissues and blood sample collection. YQ, TT, and WL designed and performed most of the molecular experiments. YQ contributed to the analysis of RNA-seq data. YC, XHL, PC, and DM provided support and technical assistance. YQ and ZH wrote the manuscript. YC provided critical revision of the manuscript. All authors read and approved the final version of the manuscript.

## Conflict of Interest

The authors declare that the research was conducted in the absence of any commercial or financial relationships that could be construed as a potential conflict of interest.

## Footnotes

1 https://rnaidesigner.invitrogen.com/rnaiexpress/

2 http://bioinformatics.psb.ugent.be/webtools/Venn/

## References

[B1] Abir R. Fisch B. (2011). Invited commentary: a single nucleotide polymorphism in BMP15 is associated with high response to controlled ovarian hyperstimulation. *Reprod. Biomed. Online* 23 77–80. 10.1016/j.rbmo.2011.03.025 21561809

[B2] Abir R. Fisch B. Johnson M. H. (2014). BMP15, fertility and the ovary. *Reprod. Biomed. Online* 29 525–526. 10.1016/j.rbmo.2014.09.007 25438664

[B3] Al-ajoury R. Kassem E. Al-halabi B. Moassess F. Al-achkar W. (2015). Investigation of some genetic variations in BMP15 accompanied with premature ovarian failure (POF) in Syrian women. *Middle East Fertil. Soc. J.* 20 91–96. 10.1016/j.mefs.2014.02.005

[B4] Baguisi A. Behboodi E. Melican D. T. Pollock J. S. Destrempes M. M. Cammuso C. (1999). Production of goats by somatic cell nuclear transfer. *Nat. Biotechnol.* 17 456–461. 10331804 10.1038/8632

[B5] Bonnet A. Servin B. Mulsant P. Mandon-Pepin B. (2015). Spatio-temporal gene expression profiling during in vivo early ovarian folliculogenesis: integrated transcriptomic study and molecular signature of early follicular growth. *PLoS One* 10:e0141482. 10.1371/journal.pone.0141482 26540452 PMC4634757

[B6] Boudreau R. L. Martins I. Davidson B. L. (2009). Artificial microRNAs as siRNA shuttles: improved safety as compared to shRNAs in vitro and in vivo. *Mol. Ther.* 17 169–175. 10.1038/mt.2008.231 19002161 PMC2834985

[B7] Braw-Tal R. McNatty K. Smith P. Heath D. Hudson N. Phillips D. (1993). Ovaries of ewes homozygous for the X-linked Inverdale gene (FecXI) are devoid of secondary and tertiary follicles but contain many abnormal structures. *Biol. Reprod.* 49 895–907. 10.1095/biolreprod49.5.895 8286585

[B8] Caixeta E. S. Sutton-McDowall M. L. Gilchrist R. B. Thompson J. G. Price C. A. Machado M. F. (2013). Bone morphogenetic protein 15 and fibroblast growth factor 10 enhance cumulus expansion, glucose uptake, and expression of genes in the ovulatory cascade during in vitro maturation of bovine cumulus-oocyte complexes. *Reproduction* 146 27–35. 10.1530/REP-13-0079 23641036

[B9] Chand A. L. Ponnampalam A. P. Harris S. E. Winship I. M. Shelling A. N. (2006). Mutational analysis of BMP15 and GDF9 as candidate genes for premature ovarian failure. *Fertil. Steril.* 86 1009–1012. 10.1016/j.fertnstert.2006.02.107 17027369

[B10] Chang H. M. Cheng J. C. Klausen C. Leung P. C. (2013). BMP15 suppresses progesterone production by down-regulating StAR via ALK3 in human granulosa cells. *Mol. Endocrinol.* 27 2093–2104. 10.1210/me.2013-1233 24140593 PMC5426602

[B11] Clement F. Monniaux D. (2013). Multiscale modelling of ovarian follicular selection. *Prog. Biophys. Mol. Biol.* 113 398–408. 10.1016/j.pbiomolbio.2012.12.005 23262160

[B12] Czauderna F. (2003). Inducible shRNA expression for application in a prostate cancer mouse model. *Nucleic Acids Res.* 31:e127. 14576327 10.1093/nar/gng127 PMC275484

[B13] Davis G. H. McEwan J. C. Fennessy P. F. Dodds K. G. McNatty K. P. Wai-Sum O. (1992). Infertility due to bilateral ovarian hypoplasia in sheep homozygous (FecX1 FecX1) for the Inverdale prolificacy gene located on the X chromosome. *Biol. Reprod.* 46 636–640. 10.1095/biolreprod46.4.636 1533540

[B14] Fabre S. Pierre A. Mulsant P. Bodin L. Di Pasquale E. Persani L. (2006). Regulation of ovulation rate in mammals: contribution of sheep genetic models. *Reprod Biol Endocrinol* 4:20. 16611365 10.1186/1477-7827-4-20 PMC1524776

[B15] Fend F. Emmert-Buck M. R. Chuaqui R. Cole K. Lee J. Liotta L. A. (1999). Immuno-LCM: laser capture microdissection of immunostained frozen sections for mRNA analysis. *Am. J. Pathol.* 154 61–66. 10.1016/s0002-9440(10)65251-0 9916919 PMC1853427

[B16] Foroughinia G. Fazileh A. Eghbalsaied S. (2017). Expression of genes involved in BMP and estrogen signaling and AMPK production can be important factors affecting total number of antral follicles in ewes. *Theriogenology* 91 36–43. 10.1016/j.theriogenology.2016.12.023 28215684

[B17] Foxcroft G. R. Hunter M. G. (1985). Basic physiology of follicular maturation in the pig. *J. Reprod. Fertil. Suppl.* 33 1–19. 3003359

[B18] Galloway S. M. McNatty K. P. Cambridge L. M. Laitinen M. P. Juengel J. L. Jokiranta T. S. (2000). Mutations in an oocyte-derived growth factor gene (BMP15) cause increased ovulation rate and infertility in a dosage-sensitive manner. *Nat. Genet.* 25 279–283. 10.1038/77033 10888873

[B19] Gilchrist R. B. Lane M. Thompson J. G. (2008). Oocyte-secreted factors: regulators of cumulus cell function and oocyte quality. *Hum. Reprod. Update* 14 159–177. 10.1093/humupd/dmm040 18175787

[B20] Gode F. Gulekli B. Dogan E. Korhan P. Dogan S. Bige O. (2011). Influence of follicular fluid GDF9 and BMP15 on embryo quality. *Fertil. Steril.* 95 2274–2278. 10.1016/j.fertnstert.2011.03.045 21496799

[B21] Golubeva Y. Salcedo R. Mueller C. Liotta L. A. Espina V. (2013). Laser capture microdissection for protein and NanoString RNA analysis. *Methods Mol. Biol.* 931 213–257. 10.1007/978-1-62703-056-4_12 23027006 PMC3766583

[B22] Grasa P. Sheikh S. Krzys N. Millar K. Janjua S. Nawaggi P. (2016). Dysregulation of follicle development in a mouse model of premature ovarian insufficiency. *Reproduction* 152 591–601. 10.1530/rep-16-0091 27581083 PMC5111581

[B23] Hashimoto O. Moore R. K. Shimasaki S. (2005). Posttranslational processing of mouse and human BMP-15: potential implication in the determination of ovulation quota. *Proc. Natl. Acad. Sci. U.S.A.* 102 5426–5431. 10.1073/pnas.0409533102 15809424 PMC556231

[B24] Hennet M. L. Combelles C. M. (2012). The antral follicle: a microenvironment for oocyte differentiation. *Int. J. Dev. Biol.* 56 819–831. 10.1387/ijdb.120133cc 23417404

[B25] Hussein T. S. Froiland D. A. Amato F. Thompson J. G. Gilchrist R. B. (2005). Oocytes prevent cumulus cell apoptosis by maintaining a morphogenic paracrine gradient of bone morphogenetic proteins. *J. Cell Sci.* 118 5257–5268. 10.1242/jcs.02644 16263764

[B26] Hussein T. S. Thompson J. G. Gilchrist R. B. (2006). Oocyte-secreted factors enhance oocyte developmental competence. *Dev. Biol.* 296 514–521. 10.1016/j.ydbio.2006.06.026 16854407

[B27] Jakimiuk A. J. Weitsman S. R. Navab A. Magoffin D. A. (2001). luteinizing hormone receptor, steroidogenesis acute regulatory protein, and steroidogenic enzyme messenger ribonucleic acids are overexpressed in thecal and granulosa cells from polycystic ovaries1. *J. Clin. Endocrinol. Metab.* 86 1318–1323. 10.1210/jcem.86.3.7318 11238527

[B28] Jiang C. Diao F. Sang Y. J. Xu N. Zhu R. L. Wang X. X. (2017). GGPP-mediated protein geranylgeranylation in oocyte is essential for the establishment of oocyte-granulosa cell communication and primary-secondary follicle transition in mouse ovary. *PLoS Genet.* 13:e1006535. 10.1371/journal.pgen.1006535 28072828 PMC5224981

[B29] Juengel J. L. Hudson N. L. Heath D. A. Smith P. Reader K. L. Lawrence S. B. (2002). Growth differentiation factor 9 and bone morphogenetic protein 15 are essential for ovarian follicular development in sheep. *Biol. Reprod.* 67 1777–1789. 10.1095/biolreprod.102.007146 12444053

[B30] Juengel J. L. Hudson N. L. Whiting L. McNatty K. P. (2004). Effects of immunization against bone morphogenetic protein 15 and growth differentiation factor 9 on ovulation rate, fertilization, and pregnancy in ewes. *Biol. Reprod.* 70 557–561. 10.1095/biolreprod.103.023333 14585806

[B31] Juengel J. L. Proctor L. E. Wearne K. Olliver D. Hudson N. L. Jensen D. (2013). Effects of immunization against androstenedione or bone morphogenetic protein 15 (BMP15) on reproductive performance in sheep. *J. Anim. Sci.* 91 5946–5953. 10.2527/jas.2012-6085 24085416

[B32] Juengel J. L. Quirke L. D. Lun S. Heath D. A. Johnstone P. D. McNatty K. P. (2011). Effects of immunizing ewes against bone morphogenetic protein 15 on their responses to exogenous gonadotrophins to induce multiple ovulations. *Reproduction* 142 565–572. 10.1530/REP-11-0126 21775423

[B33] Knox R. V. (2005). Recruitment and selection of ovarian follicles for determination of ovulation rate in the pig. *Domest. Anim. Endocrinol* 29 385–397. 10.1016/j.domaniend.2005.02.025 15998504

[B34] Liao W. X. Moore R. K. Otsuka F. Shimasaki S. (2003). Effect of intracellular interactions on the processing and secretion of bone morphogenetic protein-15 (BMP-15) and growth and differentiation factor-9. Implication of the aberrant ovarian phenotype of BMP-15 mutant sheep. *J. Biol. Chem.* 278 3713–3719. 10.1074/jbc.m210598200 12446716

[B35] Lin Z. L. Li Y. H. Xu Y. N. Wang Q. L. Namgoong S. Cui X. S. (2014). Effects of growth differentiation factor 9 and bone morphogenetic protein 15 on the in vitro maturation of porcine oocytes. *Reprod. Domest. Anim.* 49 219–227. 10.1111/rda.12254 24313324

[B36] Liu X. Liu H. Wang M. Li R. Zeng J. Mo D. (2019). Disruption of the ZBED6 binding site in intron 3 of IGF2 by CRISPR/Cas9 leads to enhanced muscle development in Liang Guang small spotted pigs. *Transgenic Res.* 28 141–150. 10.1007/s11248-018-0107-9 30488155

[B37] Long X. Peng C. Lu G. (2009). Isolation and identification of genes differentially expressed in premature luteinization granulosa cell during controlled ovarian hyperstimulation. *Fertil. Steril.* 92 1767–1771. 10.1016/j.fertnstert.2009.04.051 19616208

[B38] Luisi S. Orlandini C. Regini C. Pizzo A. Vellucci F. Petraglia F. (2015). Premature ovarian insufficiency: from pathogenesis to clinical management. *J. Endocrinol. Invest.* 38 597–603. 10.1007/s40618-014-0231-1 25596661

[B39] Luu-The V. Tremblay P. Labrie F. (2006). Characterization of type 12 17β-hydroxysteroid dehydrogenase, an isoform of type 3 17β-hydroxysteroid dehydrogenase responsible for estradiol formation in women. *Mol. Endocrinol.* 20 437–443. 10.1210/me.2005-0058 16166196

[B40] Magoffin D. A. (2005). Ovarian theca cell. *Int. J. Biochem. Cell Biol.* 37 1344–1349. 10.1016/j.biocel.2005.01.016 15833266

[B41] Mayor P. Galvez H. Guimaraes D. A. Lopez-Gatius F. Lopez-Bejar M. (2007). Serum estradiol-17beta, vaginal cytology and vulval appearance as predictors of estrus cyclicity in the female collared peccary (*Tayassu tajacu*) from the eastern Amazon region. *Anim. Reprod. Sci.* 97 165–174. 10.1016/j.anireprosci.2005.12.017 16500049

[B42] McMahon H. E. Hashimoto O. Mellon P. L. Shimasaki S. (2008). Oocyte-specific overexpression of mouse bone morphogenetic protein-15 leads to accelerated folliculogenesis and an early onset of acyclicity in transgenic mice. *Endocrinology* 149 2807–2815. 10.1210/en.2007-1550 18308851 PMC2408818

[B43] McNatty K. P. Hudson N. L. Whiting L. Reader K. L. Lun S. Western A. (2007). The effects of immunizing sheep with different BMP15 or GDF9 peptide sequences on ovarian follicular activity and ovulation rate. *Biol. Reprod.* 76 552–560. 10.1095/biolreprod.106.054361 17093201

[B44] McNatty K. P. Juengel J. L. Reader K. L. Lun S. Myllymaa S. Lawrence S. B. (2005). Bone morphogenetic protein 15 and growth differentiation factor 9 co-operate to regulate granulosa cell function. *Reproduction* 129 473–480. 10.1530/rep.1.0511 15798022

[B45] Monestier O. Servin B. Auclair S. Bourquard T. Poupon A. Pascal G. (2014). Evolutionary origin of bone morphogenetic protein 15 and growth and differentiation factor 9 and differential selective pressure between mono- and polyovulating species. *Biol. Reprod.* 91:83. 10.1095/biolreprod.114.119735 25100713

[B46] Moore R. K. Otsuka F. Shimasaki S. (2003). Molecular basis of bone morphogenetic protein-15 signaling in granulosa cells. *J. Biol. Chem.* 278 304–310. 10.1074/jbc.m207362200 12419820

[B47] Moore R. K. Shimasaki S. (2005). Molecular biology and physiological role of the oocyte factor. *BMP-15. Mol. Cell. Endocrinol.* 234 67–73. 10.1016/j.mce.2004.10.012 15836954

[B48] Mori T. (2016). “Regulatory principles of follicular development,” in *Ovarian Stimulation Protocols*, Vol. 6 eds Allahbadia G. Morimoto Y. (New Delhi: Springer), 1–16. 10.1007/978-81-322-1121-1_1

[B49] Mottershead D. G. Harrison C. A. Mueller T. D. Stanton P. G. Gilchrist R. B. McNatty K. P. (2013). Growth differentiation factor 9:bone morphogenetic protein 15 (GDF9:BMP15) synergism and protein heterodimerization. *Proc. Natl. Acad. Sci. U.S.A.* 110 E2257. 10.1073/pnas.1303459110 PMC3690902 23650403

[B50] Noguchi M. Yoshioka K. Itoh S. Suzuki C. Arai S. Wada Y. (2010). Peripheral concentrations of inhibin A, ovarian steroids, and gonadotropins associated with follicular development throughout the estrous cycle of the sow. *Reproduction* 139 153–161. 10.1530/REP-09-0018 19778995

[B51] Norris R. P. Freudzon M. Mehlmann L. M. Cowan A. E. Simon A. M. Paul D. L. (2008). Luteinizing hormone causes MAP kinase-dependent phosphorylation and closure of connexin 43 gap junctions in mouse ovarian follicles: one of two paths to meiotic resumption. *Development* 135 3229–3238. 10.1242/dev.025494 18776144 PMC2572224

[B52] Otsuka F. Yamamoto S. Erickson G. F. Shimasaki S. (2001). Bone morphogenetic protein-15 inhibits follicle-stimulating hormone (FSH) action by suppressing FSH receptor expression. *J. Biol. Chem.* 276 11387–11392. 10.1074/jbc.m010043200 11154695

[B53] Otsuka F. Yao Z. Lee T. Yamamoto S. Erickson G. F. Shimasaki S. (2000). Bone morphogenetic protein-15. Identification of target cells and biological functions. *J. Biol. Chem.* 275 39523–39528. 10.1074/jbc.m007428200 10998422

[B54] Paradis F. Novak S. Murdoch G. K. Dyck M. K. Dixon W. T. Foxcroft G. R. (2009). Temporal regulation of BMP2, BMP6, BMP15, GDF9, BMPR1A, BMPR1B, BMPR2 and TGFBR1 mRNA expression in the oocyte, granulosa and theca cells of developing preovulatory follicles in the pig. *Reproduction* 138 115–129. 10.1530/REP-08-0538 19359354

[B55] Passos M. J. Vasconcelos G. L. Silva A. W. Brito I. R. Saraiva M. V. Magalhaes D. M. (2013). Accelerated growth of bovine preantral follicles in vitro after stimulation with both FSH and BMP-15 is accompanied by ultrastructural changes and increased atresia. *Theriogenology* 79 1269–1277. 10.1016/j.theriogenology.2013.02.023 23582608

[B56] Paulini F. Melo E. O. (2011). The role of oocyte-secreted factors GDF9 and BMP15 in follicular development and oogenesis. *Reprod. Domest. Anim.* 46 354–361. 10.1111/j.1439-0531.2010.01739.x 21198974

[B57] Peng J. Li Q. Wigglesworth K. Rangarajan A. Kattamuri C. Peterson R. T. (2013). Growth differentiation factor 9:bone morphogenetic protein 15 heterodimers are potent regulators of ovarian functions. *Proc. Natl. Acad. Sci. U.S.A.* 110 E776–E785. 10.1073/pnas.1218020110 23382188 PMC3581982

[B58] Picton H. Briggs D. Gosden R. (1998). The molecular basis of oocyte growth and development. *Mol. Cell. Endocrinol.* 145 27–37. 10.1016/s0303-7207(98)00166-x 9922096

[B59] Pierre A. Peigne M. Grynberg M. Arouche N. Taieb J. Hesters L. (2013). Loss of LH-induced down-regulation of anti-Mullerian hormone receptor expression may contribute to anovulation in women with polycystic ovary syndrome. *Hum. Reprod.* 28 762–769. 10.1093/humrep/des460 23321213

[B60] Polejaeva I. A. Chen S.-H. Vaught T. D. Page R. L. Mullins J. Ball S. (2000). Cloned pigs produced by nuclear transfer from adult somatic cells. *Nature* 407 86–90. 10.1038/35024082 10993078

[B61] Pulkki M. M. Mottershead D. G. Pasternack A. H. Muggalla P. Ludlow H. van Dinther M. (2012). A covalently dimerized recombinant human bone morphogenetic protein-15 variant identifies bone morphogenetic protein receptor type 1B as a key cell surface receptor on ovarian granulosa cells. *Endocrinology* 153 1509–1518. 10.1210/en.2010-1390 22294741

[B62] Reader K. L. Heath D. A. Lun S. McIntosh C. J. Western A. H. Littlejohn R. P. (2011). Signalling pathways involved in the cooperative effects of ovine and murine GDF9+BMP15-stimulated thymidine uptake by rat granulosa cells. *Reproduction* 142 123–131. 10.1530/REP-10-0490 21474603

[B63] Reader K. L. Mottershead D. G. Martin G. A. Gilchrist R. B. Heath D. A. McNatty K. P. (2016). Signalling pathways involved in the synergistic effects of human growth differentiation factor 9 and bone morphogenetic protein 15. *Reprod. Fertil. Dev.* 28 491–498. 10.1071/RD14099 25155366

[B64] Sabel J. L. d’Alençon C. O’Brien E. K. Van Otterloo E. Lutz K. Cuykendall T. N. (2009). Maternal Interferon Regulatory Factor 6 is required for the differentiation of primary superficial epithelia in Danio and Xenopus embryos. *Dev. Biol.* 325 249–262. 10.1016/j.ydbio.2008.10.031 19013452 PMC2706144

[B65] Shackell G. Hudson N. Heath D. Lun S. Shaw L. Condell L. (1993). Plasma gonadotropin concentrations and ovarian characteristics in Inverdale ewes that are heterozygous for a major gene (FecX1) on the X chromosome that influences ovulation rate. *Biol. Reprod.* 48 1150–1156. 10.1095/biolreprod48.5.1150 8386945

[B66] Shimizu K. Nakamura T. Bayasula A. Nakanishi N. Kasahara Y. Nagai T. (2019). Molecular mechanism of FSHR expression induced by BMP15 in human granulosa cells. *J. Assist. Reprod. Genet.* 36 1185–1194. 10.1007/s10815-019-01469-y 31079267 PMC6603124

[B67] Smith P. Corrigan K. Smith T. Lundy T. Davis G. McNatty K. P. (1997). Ovarian morphology and endocrine characteristics of female sheep fetuses that are heterozygous or homozygous for the inverdale prolificacy gene (fecX1). *Biol. Reprod.* 57 1183–1192. 10.1095/biolreprod57.5.1183 9369186

[B68] Soede N. Helmond F. Kemp B. (1994). Periovulatory profiles of oestradiol, LH and progesterone in relation to oestrus and embryo mortality in multiparous sows using transrectal ultrasonography to detect ovulation. *Reproduction* 101 633–641. 10.1530/jrf.0.1010633 7966019

[B69] Soede N. M. Langendijk P. Kemp B. (2011). Reproductive cycles in pigs. *Anim. Reprod. Sci.* 124 251–258. 10.1016/j.anireprosci.2011.02.025 21397415

[B70] Spencer S. Gugliotta A. Koenitzer J. Hauser H. Wirth D. (2015). Stability of single copy transgene expression in CHOK1 cells is affected by histone modifications but not by DNA methylation. *J. Biotechnol.* 195 15–29. 10.1016/j.jbiotec.2014.12.009 25533398

[B71] Stocco D. M. (2001). StAR protein and the regulation of steroid hormone biosynthesis. *Annu. Rev. Physiol.* 63 193–213. 10.1146/annurev.physiol.63.1.193 11181954

[B72] Su Y. Wu J. He J. Liu X. Chen X. Ding Y. (2017). High insulin impaired ovarian function in early pregnant mice and the role of autophagy in this process. *Endocrine J.* 64 613–621. 10.1507/endocrj.EJ16-0494 28420820

[B73] Su Y.-Q. Sugiura K. Li Q. Wigglesworth K. Matzuk M. M. Eppig J. J. (2010). Mouse oocytes enable LH-induced maturation of the cumulus-oocyte complex via promoting EGF receptor-dependent signaling. *Mol. Endocrinol.* 24 1230–1239. 10.1210/me.2009-0497 20382892 PMC2875810

[B74] Su Y. Q. Sugiura K. Wigglesworth K. O’Brien M. J. Affourtit J. P. Pangas S. A. (2008). Oocyte regulation of metabolic cooperativity between mouse cumulus cells and oocytes: BMP15 and GDF9 control cholesterol biosynthesis in cumulus cells. *Development* 135 111–121. 10.1242/dev.009068 18045843

[B75] Sudiman J. Sutton-McDowall M. L. Ritter L. J. White M. A. Mottershead D. G. Thompson J. G. (2014). Bone morphogenetic protein 15 in the pro-mature complex form enhances bovine oocyte developmental competence. *PLoS One* 9:e103563. 10.1371/journal.pone.0103563 25058588 PMC4110049

[B76] Sugiura K. Su Y. Q. Diaz F. J. Pangas S. A. Sharma S. Wigglesworth K. (2007). Oocyte-derived BMP15 and FGFs cooperate to promote glycolysis in cumulus cells. *Development* 134 2593–2603. 10.1242/dev.006882 17553902

[B77] Sugiura K. Su Y. Q. Li Q. Wigglesworth K. Matzuk M. M. Eppig J. J. (2010). Estrogen promotes the development of mouse cumulus cells in coordination with oocyte-derived GDF9 and BMP15. *Mol. Endocrinol.* 24 2303–2314. 10.1210/me.2010-0260 21047911 PMC2999473

[B78] Sun R. Z. Lei L. Cheng L. Jin Z. F. Zu S. J. Shan Z. Y. (2010). Expression of GDF-9, BMP-15 and their receptors in mammalian ovary follicles. *J. Mol. Histol.* 41 325–332. 10.1007/s10735-010-9294-2 20857181

[B79] Wigglesworth K. Lee K. B. O’Brien M. J. Peng J. Matzuk M. M. Eppig J. J. (2013). Bidirectional communication between oocytes and ovarian follicular somatic cells is required for meiotic arrest of mammalian oocytes. *Proc. Natl. Acad. Sci. U.S.A.* 110 E3723–E3729. 10.1073/pnas.1314829110 23980176 PMC3785791

[B80] Wu X. Viveiros M. M. Eppig J. J. Bai Y. Fitzpatrick S. L. Matzuk M. M. (2003). Zygote arrest 1 (Zar1) is a novel maternal-effect gene critical for the oocyte-to-embryo transition. *Nat. Genet.* 33 187–191. 10.1038/ng1079 12539046

[B81] Wu Y. T. Tang L. Cai J. Lu X. E. Xu J. Zhu X. M. (2007). High bone morphogenetic protein-15 level in follicular fluid is associated with high quality oocyte and subsequent embryonic development. *Hum. Reprod.* 22 1526–1531. 10.1093/humrep/dem029 17347167

[B82] Yan C. Wang P. DeMayo J. DeMayo F. J. Elvin J. A. Carino C. (2001). Synergistic roles of bone morphogenetic protein 15 and growth differentiation factor 9 in ovarian function. *Mol. Endocrinol.* 15 854–866. 10.1210/me.15.6.854 11376106

[B83] Yoshino O. McMahon H. E. Sharma S. Shimasaki S. (2006). A unique preovulatory expression pattern plays a key role in the physiological functions of BMP-15 in the mouse. *Proc. Natl. Acad. Sci. U.S.A.* 103 10678–10683. 10.1073/pnas.0600507103 16818886 PMC1502291

[B84] Zhai B. Liu H. Li X. Dai L. Gao Y. Li C. (2013). BMP15 prevents cumulus cell apoptosis through CCL2 and FBN1 in porcine ovaries. *Cell Physiol. Biochem.* 32 264–278. 10.1159/000354435 23942191

