# Supplementary material for: Bone Morphogenetic Protein 15 Knockdown Inhibits Porcine Ovarian Follicular Development and Ovulation
Source: Front Cell Dev Biol. 2019 Nov 19;7:286. doi: 10.3389/fcell.2019.00286 (PMC6877722; doi:10.3389/fcell.2019.00286)
Supplement: Supplementary file 1 [file Data_Sheet_1.docx]

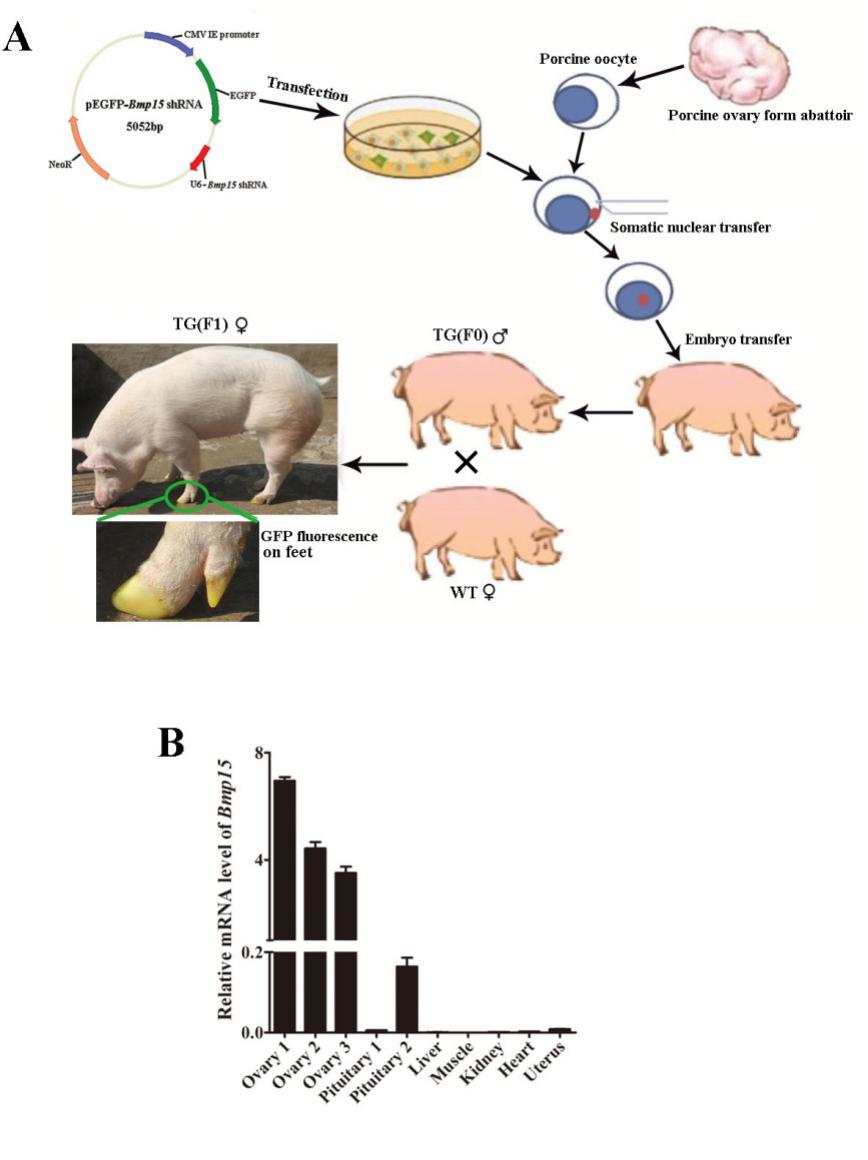


**Figure S1. Schematic diagram of generation of *Bmp15-*knockdown pig, and tissue-specific expression of *Bmp15* in gilts. (A)** Transfection of the constructed pEGFP-*Bmp15* shRNA plasmid into Yorkshire PEFs. The transfected PEFs were screened with G418 culture and [fluorescence](javascript:void(0);) selection to prepare donor cells for somatic cell nuclear transfer (SCNT). Later, we recovered the recipient porcine oocytes from abattoir-derived ovaries. SCNT and subsequent embryo transfer into Large White sow were carried out following the operating procedure of BGI Ark Biotechnology, China. We obtained two healthy neonatal F0 generation transgenic males. One TG boar was mated with wild-type sows to generate F1 gilts. Both F0 and F1 TG pigs showed visible intense GFP fluorescence on their toes while subjected to sunlight. **(B)** Tissue-specific mRNA expression profile of BMP15 in WT pigs.


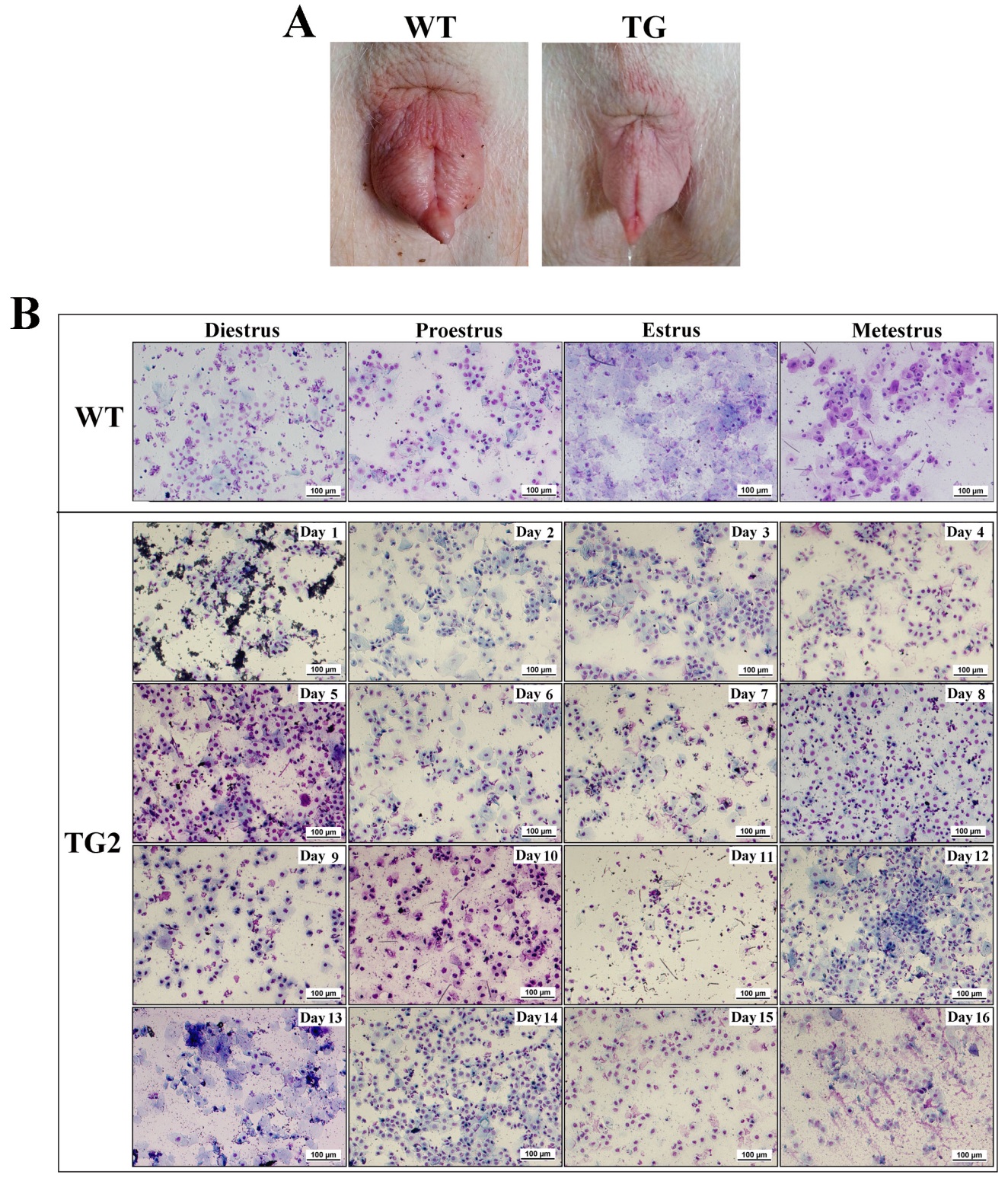


**Figure S2. TG gilts did not show obvious vulvar appearance change and typical cytologic changes through the estrous cycle.** **(A)** Vulvar appearance change (increased redness and swelling) was observed in WT gilt at estrous period, but not observed in 365-day old TG gilts, though they were induced daily by an intact mature boar starting from when they were 170 days old. **(B)** Estrous cycle was evaluated by vaginal smears cytology analysis of 365-day old gilts. It was divided into four distinct stages according to the appearance and the relative proportions of leucocytes, basal, parabasal, and superficial cells. In WT gilts, proestrus, estrus, metestrus, and diestrus stage were clearly determined through Giemsa-stained vaginal smears. However, TG gilts presented disordered estrous stages. Taking a view on the 16-day consecutive vaginal smears images of TG2, the cell type of day 13 displayed a predominance of cornified enucleate epithelial cells, which was similar to WT representative cell type of estrous stage. However, the cell types of day 14 and 15 were similar to the diestrus or proestrus stage due to the predominance of parabasal cells and few cornified superficial cells. Scale bar = 100 μm.

.


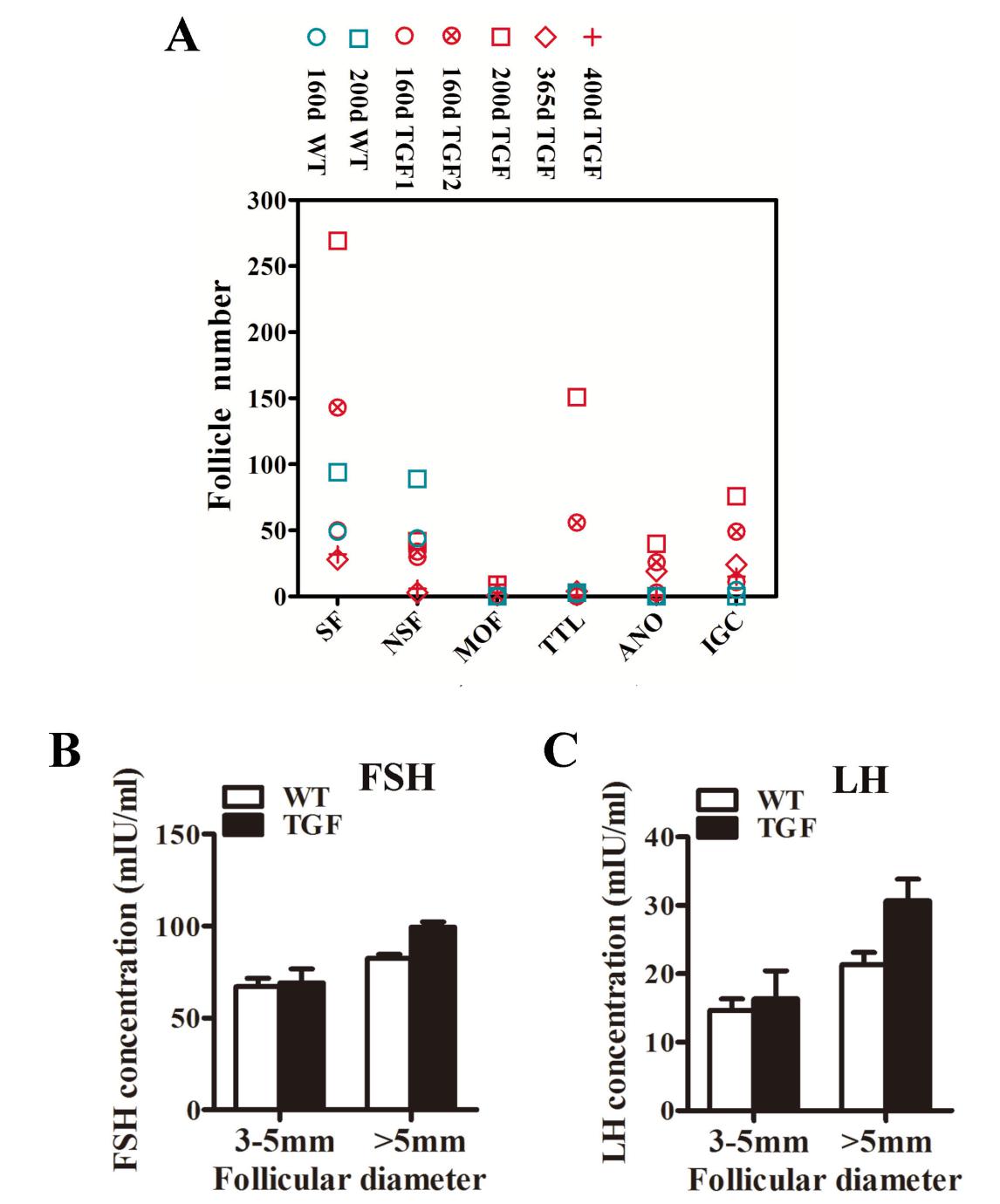


**Figure S3. Phenotypes of TGF ovaries. (A)** Statistical analysis showed less normal secondary follicles (SFs) in TGF ovaries but higher proportion of abnormal SFs. Each of three ovarian sections of two WT ovaries and five TGF ovaries were examined. These ovaries were from different gilts of ages 160– 400 days. Four types of abnormal follicular were distinguished as followings: MOF, multioocyte follicle; TTL, thickened theca and basal lamina; ANO, abnormal oocyte; IGC, irregular and degrading granular cells. NSF stands for normal secondary follicle. **(B)** The concentration of both FSH and LH **(C)** in follicular fluid was not significantly different in TGF and WT antral follicles.


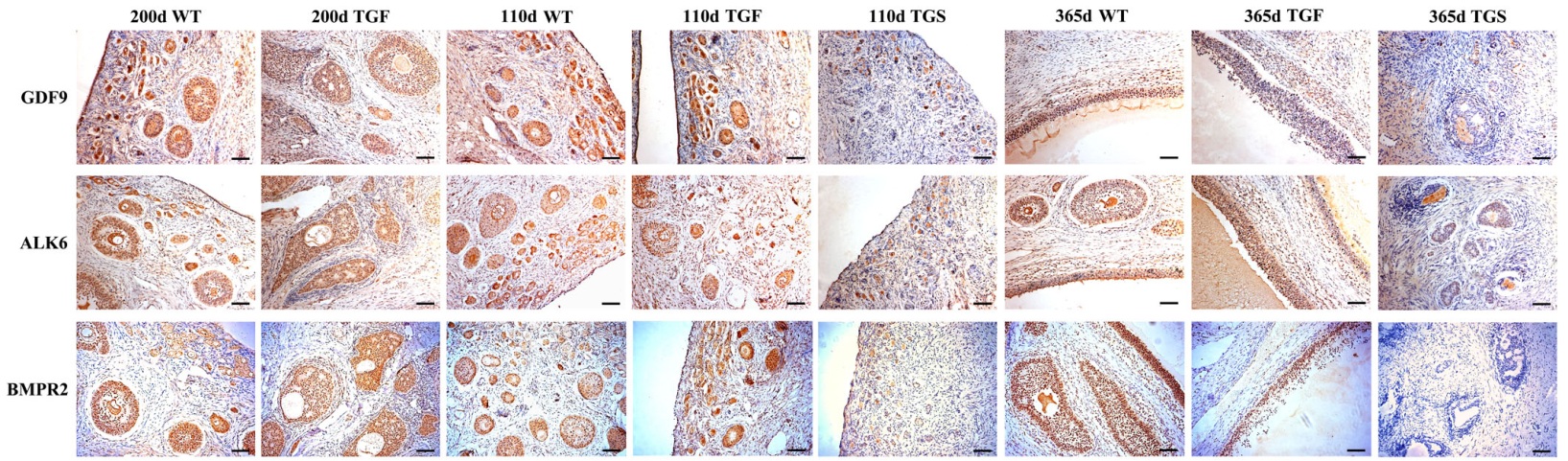


**Figure S4. Expression of GDF9, ALK6, and BMPR2 was not affected in TGF follicles.** Immunohistochemical staining showed that the expression levels of GDF9, ALK6, and BMPR2 were not significantly different in TGF and WT follicles, but significantly declined in 110 and 365-day TGS follicles. Scale bar = 100 μm.


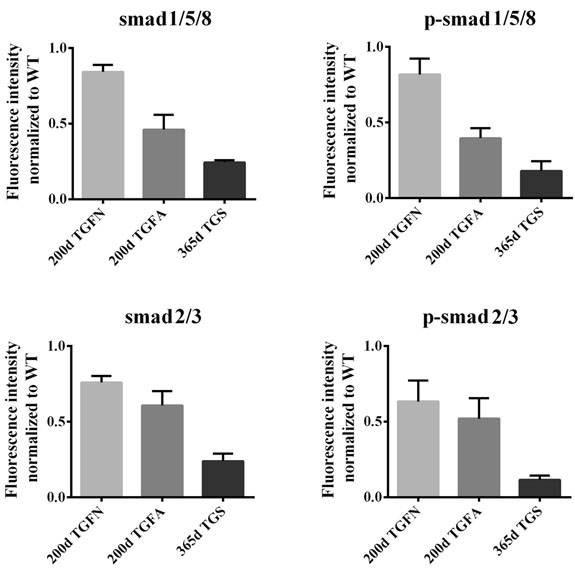


**Figure S5. Smad1/5/8 signaling transduction was more affected by BMP15 knock down in TGF abnormal follicles.** Fluorescence intensity of Smad1/5/8 signaling decreased beyond that of Smad2/3 signaling in TGF abnormal follicles, although they were both remarkably decreased in TGS follicles. Fluorescence signals were quantified by Image J software, and statistical analysis was carried out using graphpad prism software.


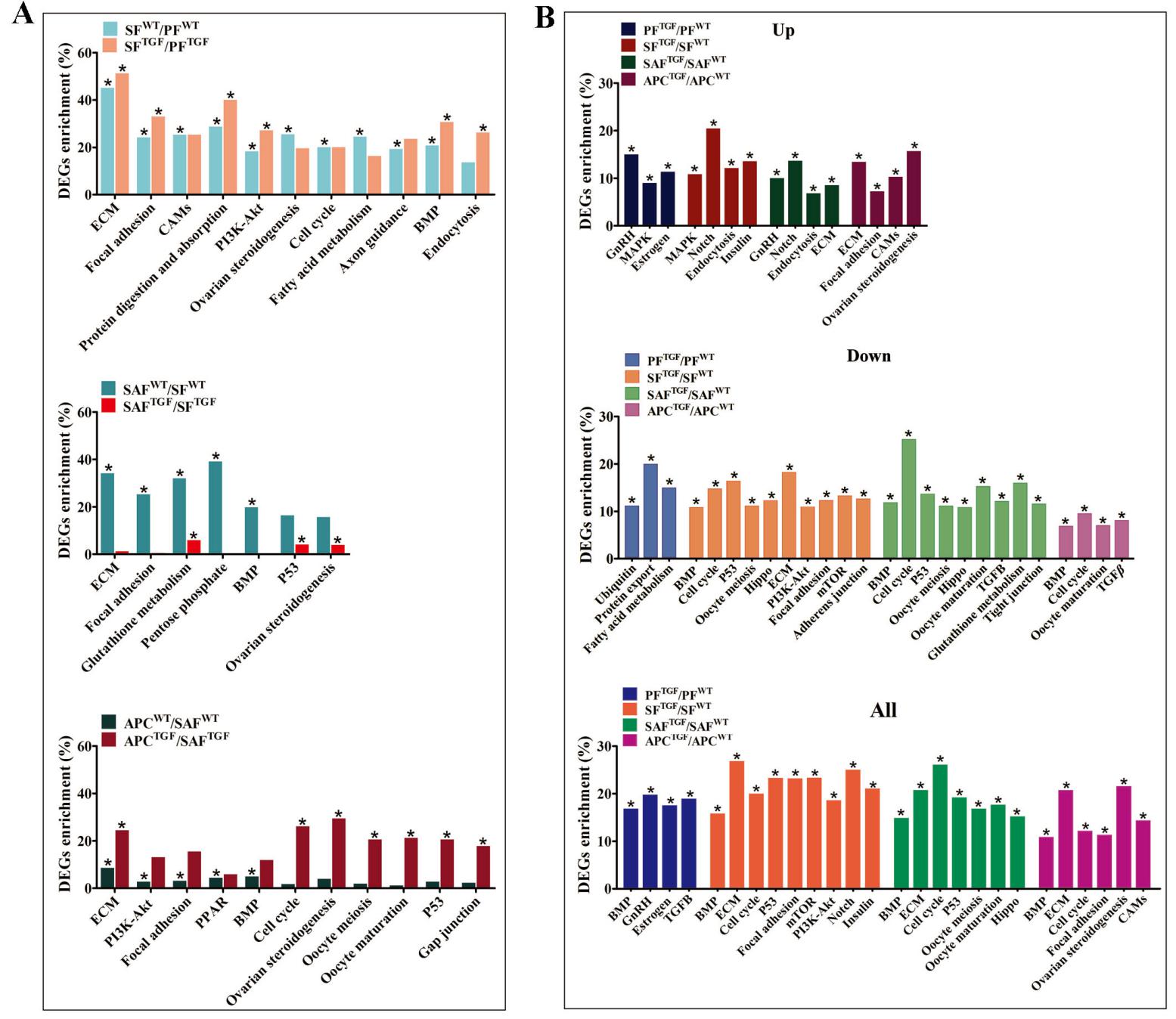


**Figure S6. Pathway analysis.** **(A)** Pathway enrichment based on intra-effect comparisons showed striking differences in the transcriptome dynamic of WT and TGF follicular development. TGF follicles showed the least number of DEGs in enriched pathways in SAF^TGF^/SF^TGF^ comparison, but the most number of DEGs in APC^TGF^/SAF^TGF^ comparison, absolutely contrary to that in WT follicles. Besides, compared with WT follicles, more significant difference in DEGs number presented in enriched pathways was found during the transition from secondary to antral follicle stage of TGF follicles rather than in the transition from primary to secondary follicle. **(B)** Pathway enrichment based on inter-effect comparisons. A total of 26 pathways were enriched in the four follicle stages, showing a tendency of down-regulated expression of pathways during TGF follicular development, especially in follicle stages beyond the primary follicle stage.


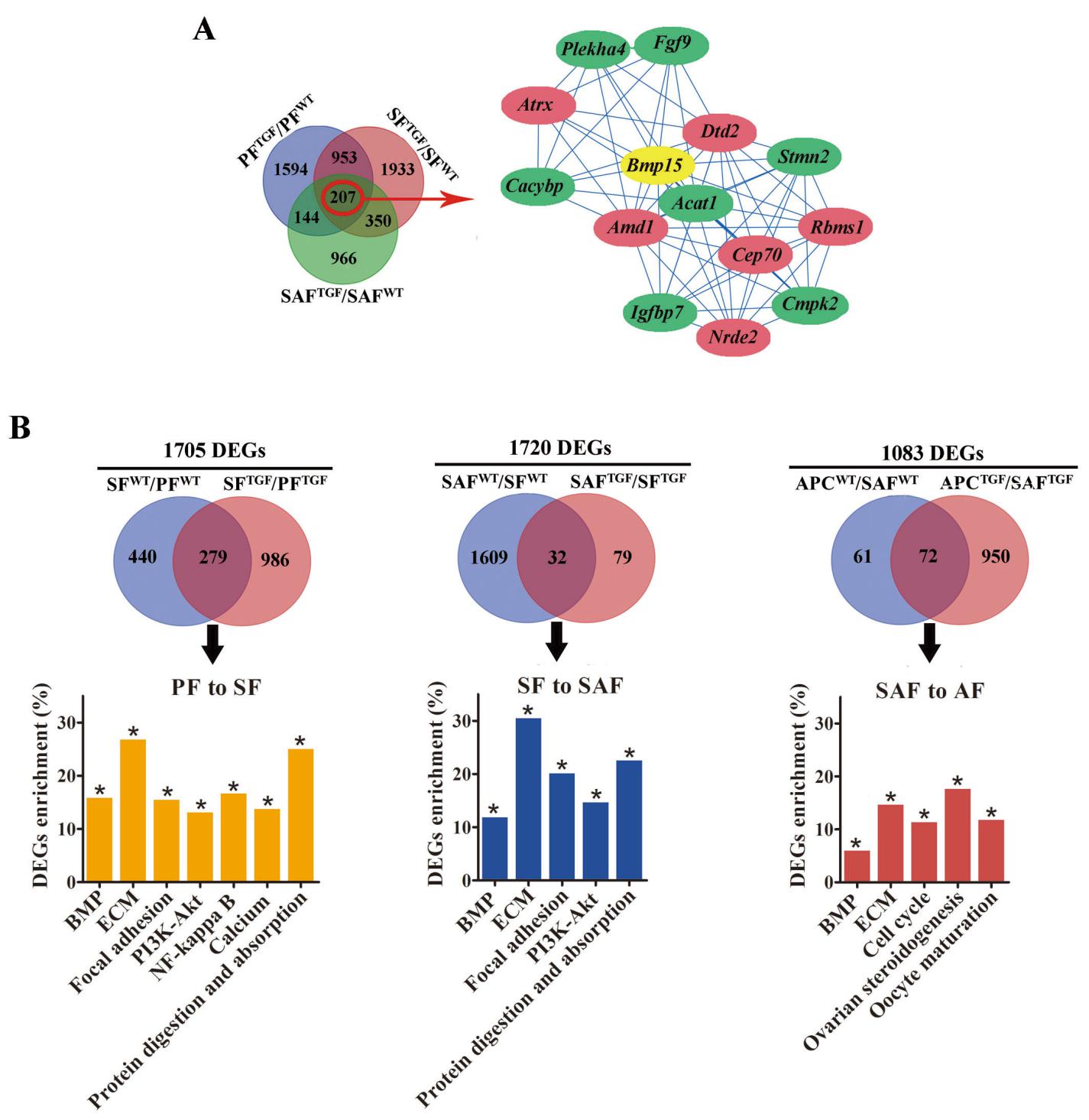


**Figure S7. Further analysis of the transcriptomic data. (A)** A correlation analysis of the DEGs predicted seven DEGs (in green ellipse) negatively correlated with *Bmp15* (correlation coefficient < -0.98), and six DEGs (in red ellipse) positively correlated with *Bmp15* (correlation coefficient > 0.98). **(B)** Further analysis of the three developmental transitions based on DEGs identification.

**Table S1 List of siRNA targeting porcine *Bmp15* gene**

| siRNAs | Sequence (5’to 3’) |
| --- | --- |
| siRNA1 F | UUGCUCCAUUAACCAAUGGTT |
| siRNA1 R | CCAUUGGUUAAUGGAGCAATT |
| siRNA2 F | GGUCCUCCUCAGCAUCAUUTT |
| siRNA2 R | AAUGAUGCUGAGGAGGACCTT |
| siRNA3 F | GGAGAUGGAUGUCACGCAATT |
| siRNA3 R | UUGCGUGACAUCCAUCUCCTT |
| siRNA4 F | CCAAGUCAGCUUCCACCAATT |
| siRNA4 R | UUGGUGGAAGCUGACUUGGTT |
| siRNA5 F | CCAACUGGGUUGGGAUCAUTT |
| siRNA5 R | AUGAUCCCAACCCAGUUGGTT |
| NC F | GGUCCUACUACGCUCCAUU TT |
| NC R | AAUGGAGCGUAGUAGGACC TT |

**Table S2 Primers for integrated plasmid detection**

| Experiment | Primer | Sequence (5’to 3’) |
| --- | --- | --- |
| PCR | shRNA-F | CTATTTCCCATGATTCCTTC |
|  | shRNA-R | ATCAGAGCAGCCGATTGT |
| RT-PCR | hU6-F | GCAGGAAGAGGGCCTATTTC |
|  | hU6-R | GTTTCGTCCTTTCCACAAGA |
| RT-PCR | TFRC-F | GAGACAGAAACTTTCGAAGC |
|  | TFRC-R | GAAGTCTGTGGTATCCAATCC |
| Southern bolt | Probe-F | GACAACCACTACCTGAGCAC |
|  | Probe-R | CGGTAAGCATATGATAGTCC |

TFRC, transferrin receptor, was used to normalized the genomic DNA.

**Table S3 Primers for qPCR detection**

| Primer | Sequence (5’to 3’) |
| --- | --- |
| *Bmp15* F | ATTAGCATCCTCCTGATTGA |
| *Bmp15* R | AACACTGAAGGCAAGAACTA |
| *Fsh* F | ATCTCCCAATCTGTCTCA |
| *Fsh* R | TAGTCCTTTCACCCATTC |
| *Lh* F | CATCACCTTTACCACCAGCATC |
| *Lh* R | GGGAAGGAGACCGTTGGGT |
| *Gdf9* F | AGACCAGCTCCAGCATCTTT |
| *Gdf9* R | GGATGGTACACCCTCAGACA |
| *Fshr* F | TTCACAGTCGCCCTCTTTCC |
| *Fshr* R | CAGCCACAGATGACCACAAA |
| *Mad2l1* F | GTTCTTCTCGTTTGGCATCA |
| *Mad2l1* R | CAAGCAAGGTAAGTCCGTAT |
| *Mkp1* F | ACCATCTGCCTCGCTTACCT |
| *Mkp1* R | GCTCCTCCTCTGCTTCACAA |
| *Cyp19* F | TCCGCAATGACTTGGGCTAC |
| *Cyp19* R | CTGGACTTTATGCACGAGGG |
| *Star* F | AAAGAACTCTATGGCTGGTA |
| *Star* R | AACAATCACTAATGGGAAAG |
| *Hsd17β1* F | CTAAGGGACTTGACGGCACA |
| *Hsd17β1* R | GGCATCCGCTATTGAATCTG |
| *Prkaa1* F | ATGGCAGAAGTTTGTAGAGC |
| *Prkaa1* R | GGAGTAGCAGTCCCTGATTT |
| *Creb1* F | GGGCAAACAAACTAAGAGGG |
| *Creb1* R | ACCAGAATGCAGACAGGTCA |
| *Npr2* F | TTTGACAGCGTTACCATTTA |
| *Npr2* R | GGGAGACCAGATACCACCAT |
| *Ccnb1* F | GAACAAGTATGCCACATCTA |
| *Ccnb1* R | AGTATTCCGAAGTTCACAAG |
| *Cdk1* F | AAAATCAGGCTAGAAAGTGA |
| *Cdk1* R | GGAGGGATAGAATCCAAGTA |

**Table S4 Weight of ovaries from gilts of different ages**

| Day | Gilt | Weight L（g） | Weight R（g） | Total weight（g） |
| --- | --- | --- | --- | --- |
| 110 | TG | 0.11 | 0.12 | 0.23 |
|  | WT | 0.82 | 0.84 | 1.66 |
| 140 | TG | 0.17 | 0.25 | 0.42 |
|  | WT | 0.55 | 0.72 | 1.27 |
| 150 | TG | 0.16 | 0.13 | 0.29 |
|  | WT | 2.41 | 2.09 | 4.5 |
| 170 | TG | 0.23 | 0.27 | 0.5 |
|  | WT | 1.0 | 0.9 | 1.9 |

L, left ovary ; R, right ovary

**Table S5 Statistical analysis of intensity of immunostaining signal for each detected factor**

| Follicle  Antibody | 200d WT | 200d TGFN | 200d TGFA | 110d TGS | 365d TGS |
| --- | --- | --- | --- | --- | --- |
| BMP15 | +++ | ++ | ++ | + | + |
| FSHR | +++ | ++ | ++ | + | + |
| LHR (preantral) | ++ | +++ | +++ | + | + |
| LHR (antral) | +++ | ++++ |  |  |  |
| 3βHSD | + |  | +++ |  | — |
| Caspase 3 | + |  | + |  | + |
| Ki67 | +++ |  | +++ |  | + |
| Smad1/5/8 | +++ | +++ | + |  | + |
| p-Smad1/5/8 | +++ | ++ | + |  | + |
| Smad2/3 | +++ | ++ | ++ |  | + |
| p-Smad2/3 | +++ | ++ | ++ |  | + |

The intensity of immunostaining signal was assessed as followings: non-detectable (—), weak but definitely positive (+), moderately positive (++), and intensely positive (+++) . TGFN, normal follicles in TGF ovaries; abnormal follicles in TGF ovaries.

**Table S7 Transcription profile of genes closely correlation with *Bmp15***

| Genes | Describe | PF^TGF^/PF^WT^ | | SF^TGF^/SF^WT^ | | SAF^TGF^/SAF^WT^ | |
| --- | --- | --- | --- | --- | --- | --- | --- |
|  |  | Log2 fold change | FDR | Log2 fold change | FDR | Log2 fold change | FDR |
| *Bmp15* | Bone morphogenetic protein 15 | -2.9 | 0.00 | -3.6 | 0.00 | -4.1 | 0.05 |
| *Amd1* | Adenosylmethionine decarboxylase 1 | -1.1 | 0.00 | -1.3 | 0.00 | -1.6 | 0.00 |
| *Cacybp* | Calcyclin binding protein | -1.4 | 0.00 | -1.1 | 0.00 | -1.0 | 0.00 |
| *Acat1* | Acetyl-CoA acetyltransferase 1 | -1.6 | 0.00 | -1.3 | 0.00 | -1.0 | 0.00 |
| *Dtd2* | D-tyrosyl-tRNA deacylase 2 | -1.1 | 0.00 | -1.5 | 0.00 | -1.7 | 0.01 |
| *Cep70* | Centrosomal protein 70 | -1.1 | 0.00 | -1.2 | 0.00 | -1.2 | 0.03 |
| *Nrde2* | Necessary for RNA interference, domain containing | 2.3 | 0.00 | 1.9 | 0.00 | 1.5 | 0.00 |
| *Plekha4* | Pleckstrin homology domain containing A4 | 1.0 | 0.02 | 1.4 | 0.00 | 1.5 | 0.00 |
| *Stmn2* | Stathmin 2 | 1.1 | 0.00 | 1.6 | 0.00 | 2.2 | 0.00 |
| *Cmpk2* | Cytidine/uridine monophosphate kinase 2 | 1.2 | 0.01 | 2.4 | 0.00 | 3.4 | 0.00 |
| *Igfbp7* | Insulin like growth factor binding protein 7 | 1.6 | 0.00 | 2.2 | 0.00 | 2.7 | 0.00 |
| *Fgf9* | Fibroblast growth factor 9 | 1.5 | 0.00 | 2.0 | 0.00 | 2.1 | 0.00 |
| *Atrx* | Chromatin remodeler | 3.3 | 0.00 | 2.1 | 0.00 | 1.6 | 0.00 |
| *Rbms1* | RNA binding motif single stranded interacting protein 1 | 2.4 | 0.00 | 1.8 | 0.00 | 1.2 | 0.00 |

**Table S8 Genes involved in oocyte meiosis**

| Gene | Describe | SF^TGF^/SF^WT^ | | | SAF^TGF^/SAF^WT^ | | | COCs^TGF^/COCs^WT^ | | |
| --- | --- | --- | --- | --- | --- | --- | --- | --- | --- | --- |
|  |  | Log2 fold change | FDR | S | Log2 fold change | FDR | S | Log2 fold change | FDR | S |
| cGMP and cAMP concentration related | | | | | | | | | | |
| *Impdh2* | Inosine monophosphate dehydrogenase 2 | -0.5 | 0.00 | ns | -0.3 | 0.73 | ns | 0.2 | 0.20 | ns |
| *Pde3a* | Phosphodiesterase 3A | 0.3 | 0.58 | ns | 1.0 | 0.59 | ns | NA | NA | ns |
| *Gpr3* | G protein-coupled receptor 3 | NA | NA | ns | NA | NA | ns | NA | NA | ns |
| *Nppc* | Natriuretic peptide C | -0.5 | 0.53 | ns | NA | NA | ns | 0.5 | 1.00 | ns |
| *Npr2* | Natriuretic peptide receptor 2 | -0.3 | 0.00 | ns | 0.0 | 0.06 | ns | 0.6 | 0.96 | ns |
| *Cx43* | Gap junction alpha-1 protein | 0.2 | 0.51 | ns | -0.5 | 0.07 | ns | 2.9 | 0.00 | s |
| *Zp3* | Zona pellucida glycoprotein 3 | -0.8 | 0.00 | ns | -1.8 | 0.00 | s | -3.0 | 0.00 | s |
| *Zp2* | Zona pellucida glycoprotein 2 | -1.1 | 0.00 | s | -2.1 | 0.00 | s | -2.8 | 0.00 | s |
| MPF related | | | | | | | | | | |
| *Cdk1* | Cyclin dependent kinase 1 | -1.6 | 0.00 | s | -2.1 | 0.00 | s | -1.7 | 0.00 | s |
| *Ccnb1* | Cyclin B1 | -1.2 | 0.00 | s | -2.9 | 0.00 | s | -2.1 | 0.00 | s |
| *Ccnb2* | Cyclin B2 | -0.8 | 0.10 | ns | -1.9 | 0.01 | s | -2.7 | 0.00 | s |
| *Wee2* | WEE1 homolog 2 | -0.8 | 0.17 | ns | -0.8 | 0.53 | ns | -3.3 | 0.00 | s |
| *Cdc25b* | Cell division cycle 25B | -0.7 | 0.00 | ns | -0.8 | 0.34 | ns | -2.3 | 0.00 | s |
| *Cdc25c* | Cell division cycle 25C | -0.4 | 0.21 | ns | -2.4 | 0.07 | ns | -3.0 | 0.00 | s |
| *Mos* | MOS proto-oncogene, serine/threonine kinase | -0.1 | 0.31 | ns | -0.8 | 0.54 | ns | -3.0 | 0.00 | s |
| APC/C-CDC20 complex regulators | | | | | | | | | | |
| *Anapc5* | Anaphase promoting complex subunit 5 | 0.1 | 0.43 | ns | -0.3 | 0.73 | ns | 2.3 | 0.00 | s |
| *Emi1* | F-box protein 5 | -1.3 | 0.00 | s | -1.4 | 0.02 | s | -2.3 | 0.00 | s |
| *Emi2* | F-box protein 43 | NA | NA | ns | NA | NA | ns | -2.7 | 0.00 | s |
| M1 division related | | | | | | | | | | |
| *Pttg1* | Pituitary tumor-transforming 1 | -1.0 | 0.00 | s | -1.7 | 0.00 | s | -2.7 | 0.00 | s |
| *Smc1b* | Structural maintenance of chromosome 1B | -2.1 | 0.00 | s | -3.1 | 0.02 |  | -2.8 | 0.00 | s |
| *Rec8* | REC8 meiotic recombination protein | 1.5 | 0.01 | s | 1.4 | 0.00 | s | 1.5 | 0.98 | ns |
| M2 division related | | | | | | | | | | |
| *Bub1* | BUB1 mitotic checkpoint serine/threonine kinase | -0.6 | 0.38 | ns | -2.5 | 0.22 | ns | -1.6 | 0.00 | s |
| *Ppp2r1b* | Protein phosphatase 2 scaffold subunit Abeta | -1.4 | 0.00 |  | -0.5 | 0.69 | ns | -1.3 | 0.00 | s |
| *Sgo1* | Shugoshin 1 | -0.1 | 0.48 | ns | -2.1 | 0.12 | ns | -1.8 | 0.00 | s |
| DNA repair and spindle stability related | | | | | | | | | | |
| *Dlgap5* | DLG associated protein 5 | -1.8 | 0.00 | s | -1.3 | 0.00 |  | -2.3 | 0.00 | s |
| *Ttk* | TTK protein kinase | -3.4 | 0.01 | s | -1.8 | 0.02 | s | -1.2 | 0.00 | s |
| *Kif18a* | Kinesin family member 18A | -2.0 | 0.04 | s | -1.8 | 0.02 | s | -2.2 | 0.00 | s |
| Anothers | | | | | | | | | | |
| *Camk2d* | Calcium/calmodulin dependent protein kinase II delta | 1.3 | 0.04 | s | 1.4 | 0.01 | s | 2.0 | 0.00 | s |
| *Mad2l1* | Mitotic spindle assembly checkpoint protein | -1.0 | 0.00 | s | -2.0 | 0.00 | s | -1.2 | 0.00 | s |
| *Slk* | STE20 like kinase | -0.5 | 0.01 | ns | -0.5 | 0.61 | ns | 1.5 | 0.00 | s |
| *Pgrma1* | progesterone receptor membrane component 1 | 0.3 | 0.48 | ns | -0.3 | 0.72 | ns | 1.3 | 0.00 | s |
| *Aurkb* | Aurora kinase B | -0.2 | 0.21 | ns | -1.1 | 0.02 | s | -2.0 | 0.00 | s |
| *Cdk2c* | Cyclin dependent kinase inhibitor 2C | -0.7 | 0.04 | ns | -1.5 | 0.00 | s | 2.3 | 0.00 | s |
| *Bmp15* | Bone morphogenetic protein 15 | -3.6 | 0.00 | s | -4.1 | 0.05 | s | -6.2 | 0.00 | s |
| *Gdf9* | Growth differentiation factor 9 | -0.9 | 0.00 | ns | -1.6 | 0.00 | s | -3.4 | 0.00 | s |
| *Chek1* | Checkpoint kinase 1 | -0.4 | 0.22 | ns | -1.2 | 0.17 | ns | -1.9 | 0.00 | s |

Red marked for significantly increase expression, Green marked for significantly decrease expression.

s, significant; ns, no significant.
